# Large scale and information effects on cooperation in public good games

**DOI:** 10.1038/s41598-019-50964-w

**Published:** 2019-10-21

**Authors:** María Pereda, Ignacio Tamarit, Alberto Antonioni, Jose A. Cuesta, Penélope Hernández, Angel Sánchez

**Affiliations:** 1 Universidad Politécnica de Madrid. Escuela Técnica Superior de Ingenieros Industriales, Departamento Ingeniería de Organización, Administración de empresas y Estadística, Madrid, Spain; 2 Unidad Mixta Interdisciplinar de Comportamiento y Complejidad Social (UMICCS), Madrid, Spain; 3 Grupo Interdisciplinar de Sistemas Complejos, Departamento de Matemáticas, Universidad Carlos III de Madrid, 28911 Leganés, Madrid, Spain; 4 Institute for Biocomputation and Physics of Complex Systems (BIFI), University of Zaragoza, 50018 Zaragoza, Spain; 5 Institute UC3M-BS for Financial Big Data (IFiBiD), Universidad Carlos III de Madrid, 28903 Getafe, Madrid, Spain; 6 ERI-CES and Departamento de Análisis Económico, Facultad de Economía, Universidad de Valencia, Avenida de los Naranjos s/n, 46022 Valencia, Spain

**Keywords:** Social evolution, Human behaviour

## Abstract

The problem of public good provision is central in economics and touches upon many challenging societal issues, ranging from climate change mitigation to vaccination schemes. However, results which are supposed to be applied to a societal scale have only been obtained with small groups of people, with a maximum group size of 100 being reported in the literature. This work takes this research to a new level by carrying out and analysing experiments on public good games with up to 1000 simultaneous players. The experiments are carried out via an online protocol involving daily decisions for extended periods. Our results show that within those limits, participants’ behaviour and collective outcomes in very large groups are qualitatively like those in smaller ones. On the other hand, large groups imply the difficulty of conveying information on others’ choices to the participants. We thus consider different information conditions and show that they have a drastic effect on subjects’ contributions. We also classify the individual decisions and find that they can be described by a moderate number of types. Our findings allow to extend the conclusions of smaller experiments to larger settings and are therefore a relevant step forward towards the understanding of human behaviour and the organisation of our society.

## Introduction

The provision of public goods has received an enormous amount of attention in the last decades, not only in experimental economics^1–3^ but in other disciplines as well^4,5^. All this research arises because of the dilemma between the individually rational decision and the collective optimal outcome: the Nash equilibrium of the game is not to contribute to the common good, while the collective optimal outcome obtains from everybody contributing as much as possible. While this is an interesting theoretical problem, it is also crucial in many real world situations. Societal challenges such as climate change mitigation^6^, ecosystem protection and sustainable exploitation^7^ or epidemic prevention^8^ can only be dealt with if many people are willing to contribute voluntarily.

In the examples we have just mentioned as well as in many others that are societally relevant, cooperation has to take place on a large or very large scale. In fact, going from two to several individuals already has serious consequences when the interaction is repeated. Two people can actually end up cooperating in a stable manner via the mechanism of reciprocity, i.e., by answering non cooperative acts with similar behaviour (e.g., tit-for-tat strategies^9^). However, three or more individuals end up cooperating less and less as time passes because reciprocal acts can not be directed only to non-contributors, and harm cooperators as well as defectors^10^. In fact, this problem of non-discriminating retaliation can be solved by directed punishment and, indeed, it is well known that when individuals can punish others separately cooperation can be sustained^11^. Notwithstanding, the second-order free rider problem^12^ and the intrinsic difficulties in monitoring other participants are serious obstacles for the punishment mechanism to work to promote cooperation.

Much less is known about what happens when the size of the group increases to the large numbers that are typical of organisational or societal endeavours, whether group size affects individual cooperative behaviour or not. The most studied paradigm for this problem is the public good game^1^ (PGG), where participants receive an endowment and decide how much of that endowment they want to contribute to a common pool. The sum of all contributions is then multiplied by a factor *r* and the resulting amount is shared equally among all participants, irrespective of their contribution. It is customary to define the marginal per-capita return (MPCR) of the PGG as the ratio of *r* to the number of group members. Following Diederich *et al*.^13^, we will consider large groups those consisting of 20 or more people and, as we will discuss below, only a handful of experimental results obtained using PGG are available. For group sizes 10 or less, experiments with university students were pioneered by Isaac *et al*.^14,15^. The available evidence shows little or no group size effects and, in addition, the effects depend on the different conditions investigated, which generally speaking change significantly from setup to setup (see, e.g., refs^13,16^ and references therein). Information effects in small groups (four people) have been considered in refs^17,18^, finding that contributions to a public good were higher when participants had explicit information of how much the others contributed to the public good.

As advanced above, experimental results on large groups of people playing PGG have been reported in only a few papers which, to the best of our knowledge, are the following ones. The first results were obtained by Isaac *et al*.^19^, finding a positive group size effect, average cooperation increased, for a marginal per-capita return (MPCR) of 0.3 but not for an MPCR of 0.75. In a more recent work, Weimann *et al*.^20^ run PGG experiments with 30 and 40 participants and MPCRs between 0.04 and 0.12, and also groups with 100 participants and MPCRs between 0.02 and 0.04. Their results show that, as far as first round contributions are concerned, small and large groups behave similarly. As the game is repeated, the contributions decay as usual and again the results are qualitatively the same across groups. Subsequently, Diederich *et al*.^13^, working with subjects from the general population in groups of 10, 40 and 100 members, found a positive group size effect, with contributions declining more slowly in the large groups. Free riding was invariant as a function of the group size though. Beyond that size there is, to the best of our knowledge, only one study^21^. Although this paper claims to report on a large-scale PGG experiment, players were recruited only in groups of 4 and informed only of the decision of one other randomly chosen player, so they did not actually participate in a large group, either directly or indirectly through the information feedback, and therefore the results do not shed light on large scale issues. Therefore, the available evidence was inconclusive, since the larger studied size remained 100 people.

In order to test experimentally the effect of group size on cooperation, we have conducted a PGG experiment with the largest group size up to date–a thousand people–as well as several experiments with 100 people for comparison. We have addressed the issue of the size dependence but, in view of the intrinsic difficulties in giving feedback to participants when the group is large, we have also analysed the influence of the way that information about group contributions is given to the participants through different experimental treatments. Our protocol has also allowed us to estimate the dropout rate in online experiments, a relevant piece of information to ascertain the effect of long decision intervals on cooperation.

Our main conclusions can be summarised as follows. We have not found any relevant size scaling comparing the results obtained with 1,000 subjects to those with 100 subjects. Thus, PGGs of 100 people seem to act as a good proxy for larger PGGs. Interestingly though, we do have found that what people contribute in a PGG very much depends on how the information on what the others are doing is presented to them. Subjects respond very differently if this information is provided through an average or through the distribution of different contributions. Finally, we have found that the dropout rate during a two-week-long experiment is less than 20%, certainly a non-negligible value that either calls for measures to mitigate it or asks for further analysis to quantify how it affects the conclusions of these experiments.

## Results

### Experimental setup

We have conducted a series of experiments on PGG. The experiments included three treatments and a control. The latter (thereafter PGG100) is a standard PGG of 100 people who contribute together to the same pool (see Methods for information about first-day dropouts). The information that each participant receives after each round is the standard one: her own contribution, her earnings in the past round, her cumulative earnings, and the average group contribution in the past rounds. The first treatment (thereafter PGG1000) is aimed to study the scaling effect and consists of a PGG with 1,000 people playing all together and contributing to the same pool. Participants receive the same information as in the control treatment. The second and third treatments study the effect of information on people’s decisions. The second treatment (thereafter PGG_HM; H stands for histogram, M stands for mean) is a PGG of 100 people, where the distribution of the other people’s contributions to the pool in the past round is provided along with the same information that participants receive in the control treatment. The third treatment (thereafter PGG_H; H stands for histogram) is identical to the second one except that people’s average contribution is omitted. We performed two repetitions with different subjects of treatments two (PGG_H and PGG_H2) and three (PGG_HM and PGG_HM2). All experiments lasted 14 days, a round per day. Thus they had 24 hours to make a decision on how much to contribute in that round, based on the information they had. In all PGG treatments, the endowment was the same, 10 points, and they had to decide how many of them (0, 2, 4, 6, 8, 10) wanted to contribute to the common pool. The Marginal Per Capita Return (MPCR) was 0.1 for all experiments, except for the PGG1000 were it was 0.01 to ensure comparison with the control treatment, preserving the ratio “multiplication factor/endowment”–that is, the pool return with respect to the endowment. The MPCR was communicated to the participants as a percentage of the group contribution, thus maintaining the MPCR constant even when people were dropping out. See Methods for further information.

### Size effects on cooperation

**Figure 1:**
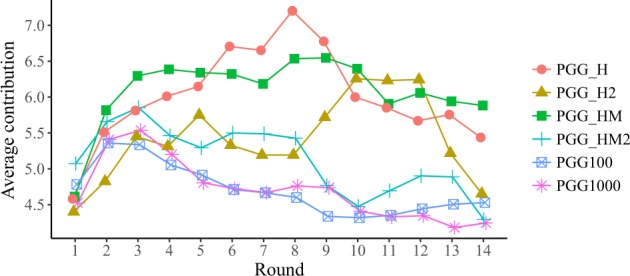
Average cooperation values per round and per treatment. Error bars are not shown because de distributions of contributions per round are not unimodal; for a representation of dispersion, we point the reader to Fig. 2. In all PGG treatments, the endowment was 10 points, and they had to decide how many of them (0, 2, 4, 6, 8, 10) wanted to contribute to the common pool. The MPCR was 0.1 for all experiments, except for the PGG1000 were it was 0.01.

We begin the discussion of our results by analysing the evolution of the average contributions to the pool on the different treatments, beginning with PGG100 and PGG1000 to focus on the effects of group size. Figure 1 shows that the average levels of cooperation of these two treatments are indistinguishable, suggesting that a group size of 100 individuals is a good representative of large groups. In the SI (Fig. S4),we represent the same information in a normalised plot, where the initial average donation of each treatment is subtracted from all subsequent round values—so that all curves start in the same zero value. Further evidence in favor of the same conclusion appears in Fig. S5, where we represent the average contributions of groups of 100 subjects randomly selected among participants in the PGG1000 treatment. As we can see, the values and evolution of the average contributions are very close to those observed both in the PGG100 and the PGG1000 experiments, reinforcing our conclusion that there are no relevant size effects up to these scales.

In order to better understand the differences between the control and the experimental treatments, it is worth exploring the evolution of the distribution of contributions per round. As we will see below, these distributions turn out to be much more informative than the simple means represented in Figs 1 and S4. We will be using heatmaps as a representation of the distribution of decisions per round.

**Figure 2:**
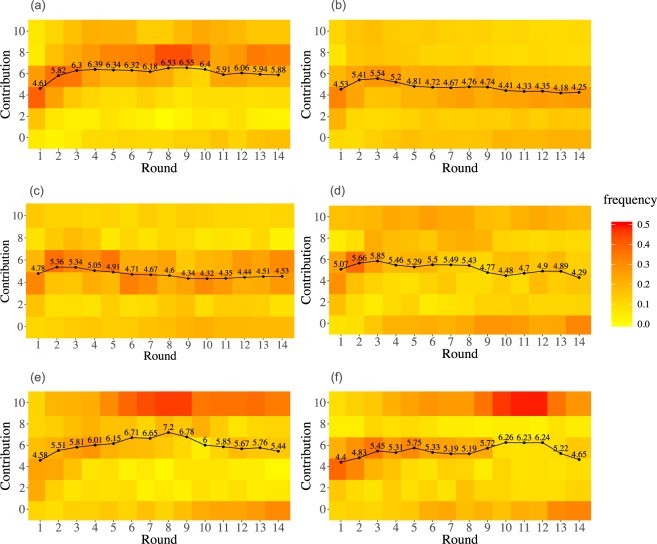
Evolution of the frequency of different decisions per round (heatmap) along with the average contribution (black line), for (**a**) PGG100, (**b**) PGG1000, (**c**) PGG_HM, (**d**) PGG_HM2, (**e**) PGG_H, (**f**) PGG_H2. In the heatmap, yellowish (redish) squares correspond to low (high) frequencies (see scale).

The first thing one can notice looking at panels a and b in Fig. 2 is that the distribution of contributions per round in PGG100 and PGG1000 are rather similar. They both are centred around the average value of the endowment, closely following the average contributions of the group (rising in the first two or three rounds and subsequently decreasing). The average contribution evolution decreases with the number of rounds, in agreement with all the previous research on PGG, albeit there is an important quantitative difference in the rate at which contributions decrease—in our experiments this decrease is slower than what is observed in the typical, small-sized PGG experiments. On the other hand, the distribution of decisions in these two treatments is unimodal, hence the mean is close to the median and to the mode, rendering the average contribution a good proxy of the general behaviour. This also holds in the randomly selected subsamples of the PGG1000 experiment (see Supplementary information for the corresponding heatmaps). As we will see below, this will not be the case when information about past behaviour is presented in a different manner.

Another fact that should be taken into account is that in our setup, decisions are made on a daily basis. When experiments are conducted in the lab, people make decisions repeatedly in relatively short time. The very different mind setups with which subjects face these decisions render a comparison with previous results particularly relevant. From the experiments available in the literature, the ones with a closer multiplication factor/endowment ratio (see Methods) to ours are those of Dietrich *et al*.^13^. The average contributions reported in that reference fall within the range of variability of our results, suggesting that their potential differences are not statistically significant. Thus we can conclude that the far longer decision interval of our experiments does not seem to have a significant impacting on the results. Normann *et al*.^22^ reached a similar conclusion in Cournot market experiments. Unfortunately, we do not have access to the data of previous experiments to perform a more quantitative comparison.

### Information effects on cooperation

We now turn to the discussion of the effect of information on both individual contributions and collective outcomes of the PGG. Along with the two treatments just discussed, where participants were informed of the mean contribution, we have also considered the effect of providing them the distribution of players’ contributions, either alone or together with the mean contribution. Figures 1 and S4 show that the contribution in the first round is close to half the endowment (46.6%), with no significant differences between treatments (see Wilcoxon-Mann-Whitney rank sum test results in Methods). Given the lack of information in the first round, this result makes sense. This value is comparable to what has been observed in previous experiments (see e.g. refs^23,24^), as it is the fact that during the first three rounds the average contributions remains at similar values irrespective of the treatment. In fact, the average contribution does not decrease monotonically from the beginning, but shows a slight increase for a few rounds (eight rounds in the PGG_H and PGG_HM treatments, and three in the rest), decreasing afterwards. This feature appears in other PGG experiments (see e.g. ref.^25^). We thus conclude that, concerning the average contributions (Fig. 1), none of the six treatments considered here exhibits very significant differences.

As it comes to the distribution of players’ contributions and players’ behaviour, there is a big difference between PGG_H and PGG100 or PGG1000 (see Fig. 2). When subjects are not being informed about the average contribution, the distribution of contributions becomes bimodal, i.e. contributions concentrate at the extreme values (donating all and donating nothing), although the average contributions are similar to those of PGG100 or PGG1000–a consequence of having an almost equal proportion of full cooperators and full defectors. The distribution of contributions is already polarised at the two extreme values by round 6, a behaviour that could be interpreted as herding.

**Figure 3:**
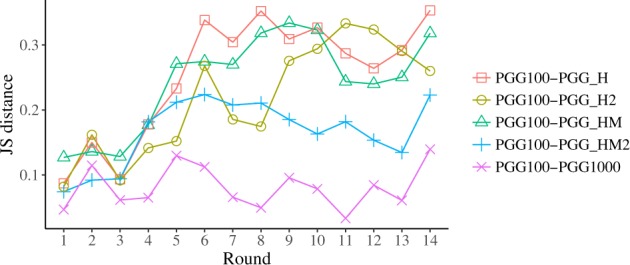
Jensen-Shannon distance of distribution of decisions per round, each pair of control-treatment.

As for PGG_HM and PGG_HM2, the average contributions in these two treatments are higher than in the control (reaching levels comparable to PGG_H), but the distribution of contributions are centred around 60–80% of the endowment rather than polarised. This modal behaviour of the contributions again suggests hearding among players. Subjects’ behaviour in the two repetitions (PGG_HM and PGG_HM2) of the treatment are different though, both in terms of average contributions (Fig. 1), and of distributions (Fig. 2 panels c and d). The average contribution in PGG_HM is closer to PGG_H and PGG_H2; on the contrary, the average contribution in PGG_HM2 is closer to PGG100 and PGG1000. If we now look at the differences between the control and the information treatments using Jensen-Shannon distance (Fig. 3) we see that PGG_H and PGG_H2 are both far from the PGG100 and PGG1000 treatments and close to each other. PGG_HM is also close to PGG_H and PGG_H2–confirming what a visual inspection of the histograms in Fig. 2 reveals–and PGG_HM2 is somewhat midway between PGG_H and PGG_H2 and PGG100 and PGG1000.

All in all, what we observe is that the way information about other people’s contributions is framed has a strong influence on individual’s behaviours. Even when information is redundant (providing the average along with the distribution) it may have unexpected effects on people’s behaviour.

### Further insights on individual behaviours

**Figure 4:**
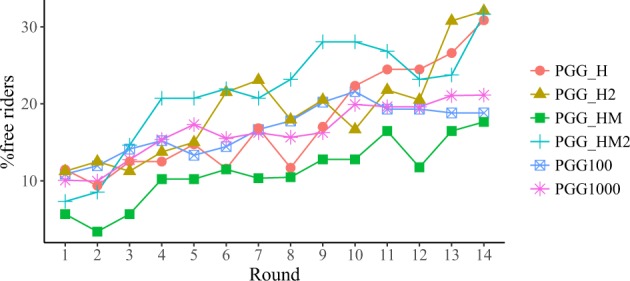
Percentage of free riders per round.

**Figure 5:**
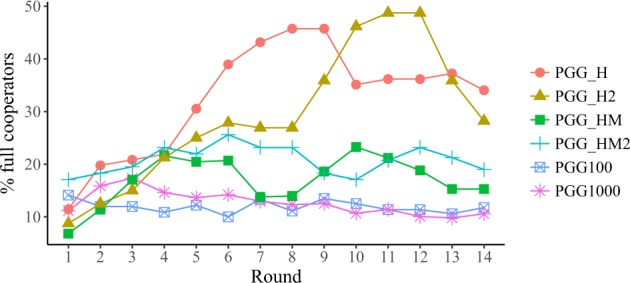
Percentage of full cooperators per round.

Having examined the collective results of the PGGs in our experiment, we now turn our focus to individual behaviours, beginning with those that are more clearly observable, namely free riders (people who contribute nothing) and full cooperators (people who contribute the full endowment). We stress that what we are discussing at this point is evolution of the fraction of people making certain decisions, but the specific individuals who made the decision at one round may not coincide with those who made the same decision at other rounds. We will come to the behaviour of individuals along the whole experiment later. Figure 4 shows the evolution of the fraction of free riders, whereas Fig. 5 depicts the evolution of the fraction of full cooperators. As expected, the fraction of free riders in the population increases with the number of rounds, at a roughly constant rate. On the contrary, the evolution of the fraction of full cooperators depends a lot on the treatment, increasing more in PGG_H and PGG_H2. Remember that in this treatment the fraction of full cooperators is a piece of information that is provided to the participants, whereas the average contribution is not. This implies that, without the explicit knowledge of the average contribution, full cooperation drags a sizeable fraction of the participants by contagion–although our results are also compatible with this being a transient, because the number of people contributing the full endowment decreases towards the end of the experiment.

Let us now focus on the choices that one individual made along the whole experiment. Individual’s behaviours show a high level of variability in their decisions along the experiment. To quantify this variability, we will refer to a subject’s behaviour as *monotonous* if at least 80% of the decisions along the experiment were the same. Table 1 shows the percentage of monotonous players in the different treatments. Note that the numbers are smaller than the percentage of free riding reported before. This simply reflects the fact that most free riders are not the same people along the experiment. On average, only 17% of the players were monotonous. The variability of people’s decisions, surprising as it may be, is similar to that reported in previous PGG experiments^19^.

**Table 1:** Percentage of monotonous players in the experiments.

PGG100	PGG1000	PGG_HM	PGG_HM2	PGG_H	PGG_H2
0.188	0.160	0.141	0.152	0.160	0.192

If we relax the condition for monotonicity, and we just look at the percentage of people who are consistently generous (they always contribute at least 80% of their endowment), we find that this percentage is higher in treatments where information about the distribution of contributions of the group is provided (see Table 2). The reverse is also true–the percentage of non-generous people (they always contribute at most 20% of their endowment) is smaller in these treatments. This suggests that informing players on how many people contribute what may be a promoter of cooperation.

**Table 2:** Percentages of generous and non-generous people per experiment.

	PGG100	PGG1000	PGG_HM	PGG_HM2	PGG_H	PGG_H2
all contributions > = 80% endowment	0.09	0.09	0.18	0.10	0.21	0.15
all contributions < = 20% endowment	0.15	0.13	0.04	0.14	0.07	0.13

To gain further insight on the effects of information, we now explore whether people’s behaviour is correlated with the mean, mode, or second most frequent decision of the group’s behaviour. In Table 3 we see that on average 37% of the subjects are correlated with the average contribution of the group (irrespective of whether this quantity is explicitly shown), and a smaller but similar percentage is correlated with the dynamics of the two most frequent decisions. It is worth noticing that on average, between 50% and 60% of the subjects did not show correlation with the group, i.e., their decisions were not correlated with the mean, mode, or second most frequent decision (Table 3, fourth row). On the other hand, correlation does not necessarily imply causation. Accordingly, we have run a Granger causality test^26^, statistical test used to determine whether one time series is useful in forecasting another time series, finding that the percentages of *Granger-caused* people are even smaller, and so the number of individuals whose decisions are neither caused or correlated with the rest of the group is even larger (Table 3, fifth row).

**Table 3:** Percentage of people whose decisions were (not) correlated or Granger-caused by the group contributions. Significance level *α* = 0.05.

	PGG100	PGG1000	PGG_HM	PGG_HM2	PGG_H	PGG_H2
Significantly correlated with the mean contribution of the group	0.3168	0.3791	0.402	0.3824	0.3137	0.4216
Significantly correlated with the modal contribution of the group	0.1881	0.2975	0.3431	0.3235	0.3137	0.402
Significantly correlated with the second most frequent contribution of the group	0.1584	0.3244	0.2451	0.2941	0.2451	0.3039
Not significantly correlated with the mean, modal or second most frequent contribution of the group	0.6436	0.5821	0.5196	0.5588	0.4608	0.5
Not Granger-caused by the group mean contribution or the modal contribution	0.6941	0.9268	0.7176	0.6975	0.7021	0.7145

Finally, we have tried an alternative approach to finding hidden patterns of behaviour or “types of people”, inspired by the existence of “phenotypes” or heuristic ways to play in different strategic situations observed in refs^27,28^. To this end, we run a hierarchical clustering algorithm for data series to group the behaviours of players. We chose the number of clusters most appropriate after exploring the dendrograms, which turned out to be three clusters for all the treatments (see dendrogram for PGG100 at S16). The three types found for PGG100 and PGG1000 are illustrated in Figs S6, S9. All three appear in both experiments although in different proportions. We will refer to these three types as people’s standard behaviours in PGGs. Basically, what we observe is: (a) a first cluster of low contributors, with an average contribution that decreases in time (low contributors); (b) a cluster of people who more or less follow the average contribution (average contributors); and (c) a final group formed by full cooperators and generous participants (high contributors). We also note that there is a large variability in individual behaviour along the experiment within each cluster (see autocorrelation plots of individual behaviours, Figs S12, S13, S14, S15, so this classification should be understood as indicating a general trend. Low contributors is the dominant behaviour, although in PGG1000 the generous participants (high contributors) are almost as numerous—although their average generosity is smaller and their behaviour is much more fluctuating than in PGG100.

The same analysis for the PGG_H treatments yields slightly different types of behaviours compared to the baseline treatments PGG100 and PGG1000 (see Figs S7, S10). The most prominent difference is the lack of a ‘follow-the-mean’ type, which is consistent with the fact that participants do not have information on the average contribution in these cases. Instead, we have a high share of participants (70%) belonging to high contributors, and around a quarter as low contributors, in accordance with the high polarisation observed in these treatments.

Finally (Figs S8, S11), in the PGG_HM experiments, the proportions of each type are different from those in the PGG_H treatments though.

We thus see that the analysis on individuals’ behaviours confirms the polarisation previously observed when the histogram of people’s contributions is provided. This is seen not only in the presence of two extreme types, but also in the smaller proportion of players who follow the average contribution in those cases–even when this average is also provided along with the histogram.

Lastly, we analyse the influence of gender on cooperation. Gender is treated as a binary variable since the recruitment platform only allowed participants to categorise themselves into two categories: male and female. When analysing the influence of gender in cooperation, we recover results observed in many other experiments on cooperation^29^, namely that females are more cooperative than males. In our experiments, females’ average levels of cooperation are higher in all treatments, except in PGG_HM (see Fig. S17). As far as individual behaviours are concerned, the cluster analysis does not shown any particular pattern correlated with either gender or age. The composition of all clusters is more or less homogeneous.

### Dropouts

As a side result of our protocol for online experiments–where subjects have 24 hours to make every decision, we now have some information on how people respond in long term behavioural experiments–a largely unexplored area of research. There is some psychological literature in Web-based experiments dealing with reducing dropouts rate by giving immediate feedback, financial incentives, personalisation^30^, by using trial rounds at the beginning of the experiment^31^, and by motivating participants^32^. There is little literature comparing the results of online experiments with the same experiments conducted in a lab. In their work about Cournot markets^22^, the authors concluded that, overall, there are no considerable differences between experiments in the lab and online experiments carried out through internet and lasting for a month. In another work^33^, the authors conducted a repeated public goods experiment with and without punishment, both online and in the lab, concluding that online data quality is adequate and reliable, but also finding higher levels of cooperation in the online setup that they hypothesised can be explained by the age and demographics of their online sample of subjects.

**Figure 6:**
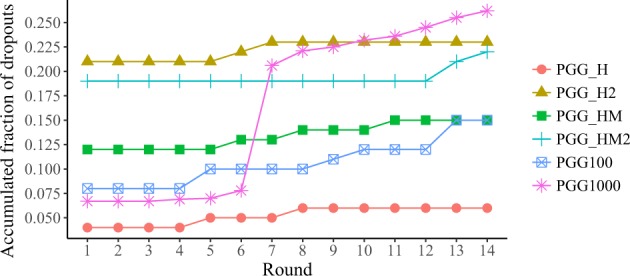
Dropouts rate per round and per treatment.

In our experimental design, we tried to incentive participation by paying the participants only at the end of the experiments, and allowing if they missed no more than just two decisions (see Methods for a more detailed description of the experimental setup). Participants who missed a third decision were banned from the experiment and received no payment whatsoever–something they were informed of from the beginning. Figure 6 shows the dropout rates (cumulative fraction of banned people) observed in all the experiments we conducted.

On average, the experiments started with a no-show fraction of 12% (participants who did not log in the first day). This no-show rate is comparable to the 10% observed in short online sessions, as reported in refs^34,35^. On the other hand, it is significantly lower than what we have observed in our laboratory experiments at Universidad Carlos III de Madrid (no-show fractions within 20–30%). Beyond the first day, the most remarkable feature of the evolution of dropouts is the high jump at round 6 in PGG1000, basically due to a technical problem (in a perfect-storm situation, a four-day holiday in Spain coincided with a blackout that disconnected the server for 2 hours). A higher fraction of subjects missed their third decisions because of this and were automatically banned from the experiment (see Methods). Leaving this incident aside, dropouts increased to a more o less constant rate, ending up at an average value of 18% on the fourteenth round (dropout rate = 0.43% per day). A constant dropout tendency was also reported by Hoerger^35^, finding no statistically significant age or gender differences.

An important potential bias introduced by dropouts might arise from a more frequent occurrence for one of the types of players than for the others. This would indirectly impact the collective behaviour. To check for this bias we compared the average contributions per round of people who did finish the experiment (Fig. S18) with that of banned people (Fig. S19). We can see that the average levels of cooperation of people who completed the experiment are practically identical to those of Fig. 1 (which average over *all* subjects, whether they completed the experiment or not). Also, the average levels of cooperation of banned subjects are comparable to those of the rest of the participants, except for the case of PGG_HM in rounds 8–10. A more detailed description of strategies played by people who dropped out can be found at Fig. S19. We can conclude then that average levels of cooperation in our experiments are only weakly (if at all) influenced by the dropouts, and that–at least in our case–the online protocol serves well the purpose of handling groups that are too large for a typical laboratory.

## Discussion

In this paper we have reported the results of experiments on PGGs in groups of 100 and 1000 participants. We have been able to achieve this participation record by designing an online protocol that allows people to interact synchronously. To that purpose, rounds of PGG lasted 24 hours, so that participation in the experiment does not interfere with participant’s daily life. The price we have to pay is to run the experiment for two weeks and, as a consequence, to endure a small fraction of dropouts. In terms of collective behaviour, all our treatments show a smaller amount of free riders than that reported in ref.^19^, where the percentage of free riders is 35–40% in groups of 100 people. As for the amount of full cooperators, the percentage found in this reference (15–20%) is comparable to what we see in the PGG_HM treatments, higher than that observed in the control and PGG100 treatment, and lower than what we found in our PGG_H treatments–where we provide the same sort of information than ref.^19^.

Our purpose in carrying out these experiments was twofold. First, we wanted to explore the large scale behaviour of PGGs beyond what was known to date. In this regard, the analysis of our results allows us to conclude that there are no relevant differences between the experiments with 100 and 1000 subjects, at least as far as collective behaviour is concerned. Our second goal was to study how subjects respond to different information conditions. Importantly, we have found that while the collective behaviour is not very different when the information is the mean or the distribution of contributions, the individual behaviour is very different, with the effect of information about distributions being the polarisation towards the extreme, i.e., contributing all or nothing. This relates to previous experimental findings in ref.^18^, where they found that individuals with a high propensity to contribute tend to imitate the highest contributor more often. We stress that this finding points out the necessity to go beyond global magnitudes to characterise PGG experiments, and also hints of consequences that can be applied to real life contexts; thus, in a social situation where it is required to foster cooperation, the effect of providing one type of information or another may lead to egalitarian contributions, clustered about the mean, or to highly different contributions, with part of the population free riding of large efforts of other part. Given that many societal challenges involve cooperation among even larger groups of people, and that individuals involved in those challenges cannot possibly monitor everybody else, this calls for further research on informational effects from the viewpoint of individual behaviours. Another finding of our work about individual behaviours is that it appears that, roughly speaking, there are only a few main types of players, namely low contributors, high contributors, and average contributors, this last group being replaced when information on distributions is available by another with less clear behaviour (but much smaller in fraction). This is further indication of the polarising effect of providing data on contribution distributions to the participants.

Finally, it is worth noting that we have developed an online protocol that allows us to tackle large groups and, for the first time to our knowledge, without requiring to recruiting workers from Amazon Mechanical Turk (AMT) but rather using our own volunteer database. In this respect, engaging people in long term experiments and reducing dropouts rate are still challenges that needs to be addressed: while the percentage of people leaving the experiment without finishing is comparable to the one of no-shows at physical laboratory, we envisage that further incentives, such as a larger payment to a player chosen by a lottery, for instance, may further reduce the attrition of the group. Further research is needed in this direction as well as to explore the possibility of reaching even larger experimental scales.

## Methods

### Experimental setup

To test the effects of group size on cooperation, we performed two treatments, PGG100 and PGG1000. For the PGG100 we recruited 100 participants and 8 of them did not show up the first day. For the PGG1000 we recruited 1004, and 71 did not show up. For the study of information effects, we conducted two treatments: PGG_HM and PGG_H (both with two repetitions, PGG_HM and PGG_HM2 (101 people recruited in each, 13 and 19 people did not show up the first day respectively); PGG_H and PGG_H2 (101 people recruited in each, 5 and 21 people did not show up the first day respectively).

The experiments were conducted in March 2017 (PGG100), April-May 2017 (PGG1000), June 2017 (PGG_HM and PGG_H), and September 2017 (PGG_HM2 and PGG_H2). Participants were Spanish volunteers from the IBSEN pool of subjects^36^. The percentage of females and ranges of age of participants is shown in Table 4. All participants in the experiment signed an informed consent to participate. In agreement with the Spanish Law for Personal Data Protection, no association was ever made between their real names and the results. This procedure was checked and approved by the Ethics Committee of Universidad Carlos III de Madrid, the institution handling the funding for the experiment, and the experiment was subsequently carried out in accordance with the approved guidelines and regulations.

**Table 4:** Percentage of female and ranges of age of participants.

Treatment	PGG100	PGG1000	PGG_HM	PGG_HM2	PGG_H	PGG_H2
% Females	54%	61.1%	70.3%	65.3%	63%	55.4%
Age range	20–54	19–69	20–74	18–64	19–61	18–60

The experiments were implemented in IBSEN-oTree^36^. In the four treatments, people played online though a web browser in a computer, tablet or mobile phone. People played online for 14 days (one round per day), but they did not know in advance there were 14 rounds to avoid last round defection, they just knew the experiment would finish at most after 20 days. People had 24 hours to make their decisions, and they saw a timer in the upper part of the page that showed the remaining time until next round. They were informed if they missed to play some day with a text in colour in the same page. Subjects were allowed to miss no more than two decisions. Missing decisions were handled by the code, which repeated their previous choice 80% of the times, and randomly increased or decreased by 1 point the remaining 20%. Whatever the case, the decision was marked as not made by the subject. If subjects missed a third decision they were banned from the experiment and deleted from our volunteer database. The experiment then continued with one less participant (note that our PGGs are defined by producing a percentage of the pool for every participant, so changing the number of subjects would no affect the pool return). Subjects that did not make the first decision were banned from the very beginning.

Participants had to decide to contribute points (experimental currency unit) and at the end of the experiment were notified of the conversion rate from points to euros, and also the quantity the had earned. Only participants who completed the experiment were paid, and they were informed about this and the banning policy in the instructions (see Instructions in the Supporting Information). They were paid a participation fee of 5 euros, and earned an average payment of 5 more euros for their decisions. Payments were done to their email addresses through PayPal after the experiment was finished. The total number of participants that completed the experiments was 1,159, with an average earning of 10 euros.

In all PGG treatments, the endowment was the same, 10 points, and they had to decide how many of them (0, 2, 4, 6, 8, 10) wanted to contribute to the common pool. The MPCR was 0.1 for all experiments, except for the PGG1000 were it was 0.01 to ensure comparison with the control treatment, preserving the ratio “multiplication factor/endowment”–that is, the pool return with respect to the endowment. The MPCR was communicated to the participants as a percentage of the group contribution, thus maintaining the MPCR constant even when people were dropping out.

Regarding the information that participants received on past rounds (after round two) there were differences among treatments (by definition). In the control treatment (PGG100) and in PGG1000, the information that each participant received a the end of the round was the standard in this experiments: her contribution and her earnings in the past round, her cumulative earnings, and the average contribution of the group in the past rounds. Average contributions were shown by means of a graph (see the screenshots provided in the Supplementary Information). In PGG_HM, participants were also informed about the distribution of contributions to the pool of the whole group in the past round. In PGG_H, the average contribution of the group in the past round was omitted, so that people could see the distribution of contributions but had not direct access to the average contribution (although they could easily compute it themselves). In both PGG_HM and PGG_H, the distributions of contributions were shown verbally (see Supplementary Information) instead of by means of a graph.

Participants were reminded to make their decisions every round through an email. The content of the email also showed their contribution and their earnings in the past round, their cumulative earnings, and the average contribution of the group in the past round (except for PGG_H were this average was not reported). They were also recalled any missing past decision.

At the beginning of the experiments, participants could read the instructions, and immediately afterwards they had to complete three control questions to make sure they understood the experiment. The instructions were also available during the whole experiment, both in the decision page and in the result pages.

The main limitation of this study is the reduced number of replications of each experiment. Budget limitations preclude to replicate large scale experiments as one normally does with the small-scale ones, so this is an inherent limitation of this kind of studies. This limitation will be circumvented when–as it is desirable in experimental sciences–other groups replicate the experiment. The same can be said about the exploration of MPCR values or the number of rounds of the experiment–which in any case should remain low, given the long duration of the experiment. Further research is needed in order to cover these limitations.

### Wilcoxon-Mann-Whitney rank sum test for the inequality of averages in first round

To test whether the the average contributions in the first round of the treatments are statistically different from the control treatment, we run a Wilcoxon-Mann-Whitney rank sum test^37^. We can not conclude that in the first round all treatments are not statistically different (we cannot reject the null hypothesis of the Wilcoxon-Mann-Whitney rank sum test, all p-values greater than 0.4), which makes sense given that in the first decision participants had no information about which feedback players would have after making their choices.

### Clustering

In order to cluster the behaviour of participants during the experiments, we applied a hierarchical clustering to the time series of decisions (contribution per round). Since we cannot work with series with missing values due to dropouts, we focus this analysis only on the participants who made all decisions. Before applying hierarchical clustering, we had to measure time series similarity. We did that using a Euclidean distance between data series (implemented in the R package *dtw*), rather than using DTW (Dynamic Time Warping) distance, because this latter metric aligns temporal series that vary in speed, but for our purpose, the time and speed of behavioural decisions are key to distinguish behaviours. After applying hierarchical clustering with complete linkage method (also using the R package *hclust*), we inspected the dendrograms and selected the number of clusters for each experimental treatment in such a way that they were kept at a minimum while at the same time we made sure that they contained at least a 10% of the series. The results of the clustering analysis is shown in the results section.

### Accession codes

Data is available in an structured way at Zenodo public repository with DOI http://doi.org/10.5281/zenodo.2590685.

## Supplementary information

Supplementary Information
