# Supplementary material for: Large scale and information effects on cooperation in public good games
Source: Sci Rep. 2019 Oct 21;9:15023. doi: 10.1038/s41598-019-50964-w (PMC6803689; doi:10.1038/s41598-019-50964-w)
Supplement: Supplementary file 1 — Supplementary Information [file 41598_2019_50964_MOESM1_ESM.pdf]

Supplementary Information

Large scale and information effects on cooperation in public good games

María Pereda[1,2,*], Ignacio Tamarit[2,3], Alberto Antonioni[3], Jose A. Cuesta[2,3,4,5], Penélope Hernández[2,6], and Angel Sánchez[2,3,4,5]

[1]Universidad Politécnica de Madrid. Departamento Ingeniería de Organización, Administración de empresas y Estadística, Madrid, Spain

[2]Unidad Mixta Interdisciplinar de Comportamiento y Complejidad Social (UMICCS), Spain

[3]Grupo Interdisciplinar de Sistemas Complejos, Departamento de Matemáticas, Universidad Carlos III de Madrid, 28911 Leganés, Madrid, Spain

[4]Institute for Biocomputation and Physics of Complex Systems (BIFI), University of Zaragoza, 50018 Zaragoza, Spain

[5]Institute UC3M-BS for Financial Big Data (IFiBiD), Universidad Carlos III de Madrid, 28903 Getafe, Madrid, Spain

[6]ERI-CES and Departamento de Análisis Económico, Facultad de Economía, Universidad de Valencia, Avenida de los Naranjos s/n, 46022 Valencia, Spain

[*]Corresponding author: mariaperedagarcia@gmail.com

August 26, 2019

Contents

1 Experimental instructions **3**

2 Supplementary Figures **6**

1 Experimental instructions

Participants were given the following instructions .....

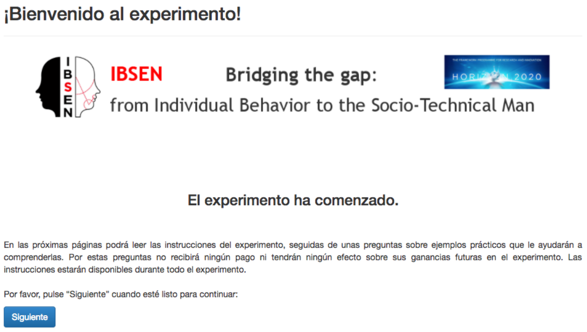

¡Bienvenido al experimento!

IBSEN Bridging the gap: from Individual Behavior to the Socio-Technical Man

HORIZON 2020

El experimento ha comenzado.

En las próximas páginas podrá leer las instrucciones del experimento, seguidas de unas preguntas sobre ejemplos prácticos que le ayudarán a comprenderlas. Por estas preguntas no recibirá ningún pago ni tendrán ningún efecto sobre sus ganancias futuras en el experimento. Las instrucciones estarán disponibles durante todo el experimento.

Por favor, pulse "Siguiente" cuando esté listo para continuar:

Siguiente

(a) Welcome screen

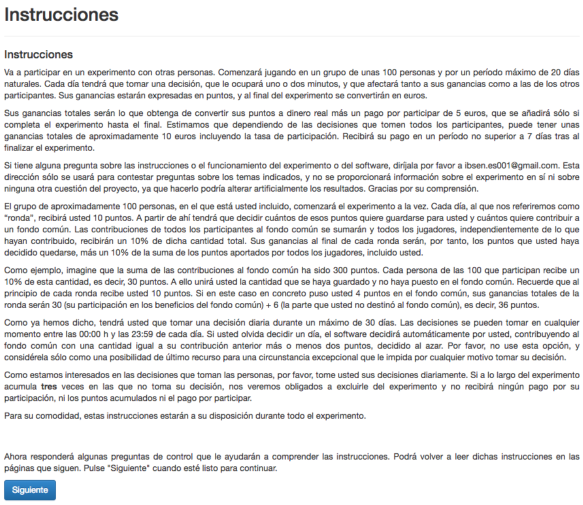

Instrucciones

Instrucciones

Va a participar en un experimento con otras personas. Comenzará jugando en un grupo de unas 100 personas y por un período máximo de 20 días naturales. Cada día tendrá que tomar una decisión, que le ocupará uno o dos minutos, y que afectará tanto a sus ganancias como a las de los otros participantes. Sus ganancias estarán expresadas en puntos, y al final del experimento se convertirán en euros.

Sus ganancias totales serán lo que obtenga de convertir sus puntos a dinero real más un pago por participar de 5 euros, que se añadirá sólo si completa el experimento hasta el final. Estimamos que dependiendo de las decisiones que tomen todos los participantes, puede tener unas ganancias totales de aproximadamente 10 euros incluyendo la tasa de participación. Recibirá su pago en un período no superior a 7 días tras al finalizar el experimento.

Si tiene alguna pregunta sobre las instrucciones o el funcionamiento del experimento o del software, diríjala por favor a ibsen.es001@gmail.com. Esta dirección sólo se usará para contestar preguntas sobre los temas indicados, y no se proporcionará información sobre el experimento en sí ni sobre ninguna otra cuestión del proyecto, ya que hacerlo podría alterar artificialmente los resultados. Gracias por su comprensión.

El grupo de aproximadamente 100 personas, en el que está usted incluido, comenzará el experimento a la vez. Cada día, al que nos referiremos como "ronda", recibirá usted 10 puntos. A partir de ahí tendrá que decidir cuántos de esos puntos quiere guardarse para usted y cuántos quiere contribuir a un fondo común. Las contribuciones de todos los participantes al fondo común se sumarán y todos los jugadores, independientemente de lo que hayan contribuido, recibirán un 10% de dicha cantidad total. Sus ganancias al final de cada ronda serán, por tanto, los puntos que usted haya decidido quedarse, más un 10% de la suma de los puntos aportados por todos los jugadores, incluido usted.

Como ejemplo, imagine que la suma de las contribuciones al fondo común ha sido 300 puntos. Cada persona de las 100 que participan recibe un 10% de esta cantidad, es decir, 30 puntos. A ello unirá usted la cantidad que se haya guardado y no haya puesto en el fondo común. Recuerde que al principio de cada ronda recibe usted 10 puntos. Si en este caso en concreto puso usted 4 puntos en el fondo común, sus ganancias totales de la ronda serán 30 (su participación en los beneficios del fondo común) + 6 (la parte que usted no destinó al fondo común), es decir, 36 puntos.

Como ya hemos dicho, tendrá usted que tomar una decisión diaria durante un máximo de 30 días. Las decisiones se pueden tomar en cualquier momento entre las 00:00 h y las 23:59 de cada día. Si usted olvida decidir un día, el software decidirá automáticamente por usted, contribuyendo al fondo común con una cantidad igual a su contribución anterior más o menos dos puntos, decidido al azar. Por favor, no use esta opción, y considérela sólo como una posibilidad de último recurso para una circunstancia excepcional que le impida por cualquier motivo tomar su decisión.

Como estamos interesados en las decisiones que toman las personas, por favor, tome usted sus decisiones diariamente. Si a lo largo del experimento acumula **tres** veces en las que no toma su decisión, nos veremos obligados a excluirle del experimento y no recibirá ningún pago por su participación, ni los puntos acumulados ni el pago por participar.

Para su comodidad, estas instrucciones estarán a su disposición durante todo el experimento.

Ahora responderá algunas preguntas de control que le ayudarán a comprender las instrucciones. Podrá volver a leer dichas instrucciones en las páginas que siguen. Pulse "Siguiente" cuando esté listo para continuar.

Siguiente

(b) Instructions screen

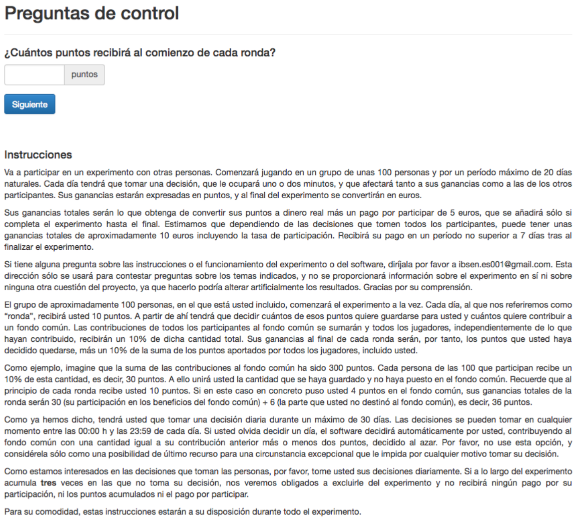

Preguntas de control

¿Cuántos puntos recibirá al comienzo de cada ronda?

puntos

Siguiente

Instrucciones

Va a participar en un experimento con otras personas. Comenzará jugando en un grupo de unas 100 personas y por un período máximo de 20 días naturales. Cada día tendrá que tomar una decisión, que le ocupará uno o dos minutos, y que afectará tanto a sus ganancias como a las de los otros participantes. Sus ganancias estarán expresadas en puntos, y al final del experimento se convertirán en euros.

Sus ganancias totales serán lo que obtenga de convertir sus puntos a dinero real más un pago por participar de 5 euros, que se añadirá sólo si completa el experimento hasta el final. Estimamos que dependiendo de las decisiones que tomen todos los participantes, puede tener unas ganancias totales de aproximadamente 10 euros incluyendo la tasa de participación. Recibirá su pago en un período no superior a 7 días tras al finalizar el experimento.

Si tiene alguna pregunta sobre las instrucciones o el funcionamiento del experimento o del software, diríjala por favor a ibsen.es001@gmail.com. Esta dirección sólo se usará para contestar preguntas sobre los temas indicados, y no se proporcionará información sobre el experimento en sí ni sobre ninguna otra cuestión del proyecto, ya que hacerlo podría alterar artificialmente los resultados. Gracias por su comprensión.

El grupo de aproximadamente 100 personas, en el que está usted incluido, comenzará el experimento a la vez. Cada día, al que nos referiremos como "ronda", recibirá usted 10 puntos. A partir de ahí tendrá que decidir cuántos de esos puntos quiere guardarse para usted y cuántos quiere contribuir a un fondo común. Las contribuciones de todos los participantes al fondo común se sumarán y todos los jugadores, independientemente de lo que hayan contribuido, recibirán un 10% de dicha cantidad total. Sus ganancias al final de cada ronda serán, por tanto, los puntos que usted haya decidido quedarse, más un 10% de la suma de los puntos aportados por todos los jugadores, incluido usted.

Como ejemplo, imagine que la suma de las contribuciones al fondo común ha sido 300 puntos. Cada persona de las 100 que participan recibe un 10% de esta cantidad, es decir, 30 puntos. A ello unirá usted la cantidad que se haya guardado y no haya puesto en el fondo común. Recuerde que al principio de cada ronda recibe usted 10 puntos. Si en este caso en concreto puso usted 4 puntos en el fondo común, sus ganancias totales de la ronda serán 30 (su participación en los beneficios del fondo común) + 6 (la parte que usted no destinó al fondo común), es decir, 36 puntos.

Como ya hemos dicho, tendrá usted que tomar una decisión diaria durante un máximo de 30 días. Las decisiones se pueden tomar en cualquier momento entre las 00:00 h y las 23:59 de cada día. Si usted olvida decidir un día, el software decidirá automáticamente por usted, contribuyendo al fondo común con una cantidad igual a su contribución anterior más o menos dos puntos, decidido al azar. Por favor, no use esta opción, y considérela sólo como una posibilidad de último recurso para una circunstancia excepcional que le impida por cualquier motivo tomar su decisión.

Como estamos interesados en las decisiones que toman las personas, por favor, tome usted sus decisiones diariamente. Si a lo largo del experimento acumula **tres** veces en las que no toma su decisión, nos veremos obligados a excluirle del experimento y no recibirá ningún pago por su participación, ni los puntos acumulados ni el pago por participar.

Para su comodidad, estas instrucciones estarán a su disposición durante todo el experimento.

(c) Preliminar control question example

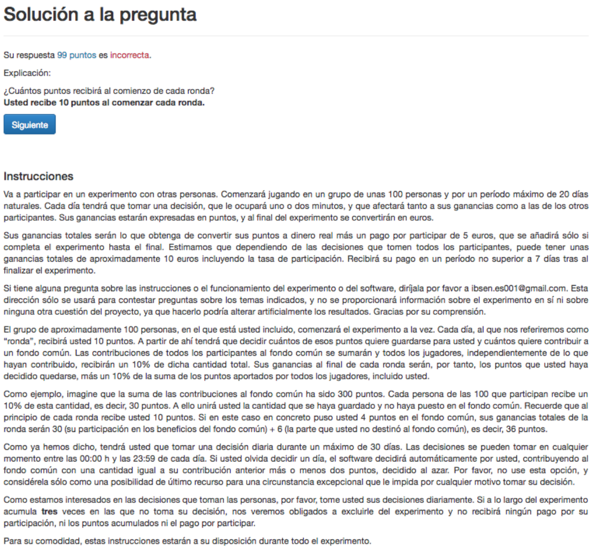

Solución a la pregunta

Su respuesta 99 puntos es incorrecta.

Explicación:

¿Cuántos puntos recibirá al comienzo de cada ronda?

Usted recibe 10 puntos al comenzar cada ronda.

Siguiente

Instrucciones

Va a participar en un experimento con otras personas. Comenzará jugando en un grupo de unas 100 personas y por un período máximo de 20 días naturales. Cada día tendrá que tomar una decisión, que le ocupará uno o dos minutos, y que afectará tanto a sus ganancias como a las de los otros participantes. Sus ganancias estarán expresadas en puntos, y al final del experimento se convertirán en euros.

Sus ganancias totales serán lo que obtenga de convertir sus puntos a dinero real más un pago por participar de 5 euros, que se añadirá sólo si completa el experimento hasta el final. Estimamos que dependiendo de las decisiones que tomen todos los participantes, puede tener unas ganancias totales de aproximadamente 10 euros incluyendo la tasa de participación. Recibirá su pago en un período no superior a 7 días tras al finalizar el experimento.

Si tiene alguna pregunta sobre las instrucciones o el funcionamiento del experimento o del software, diríjala por favor a ibsen.es001@gmail.com. Esta dirección sólo se usará para contestar preguntas sobre los temas indicados, y no se proporcionará información sobre el experimento en sí ni sobre ninguna otra cuestión del proyecto, ya que hacerlo podría alterar artificialmente los resultados. Gracias por su comprensión.

El grupo de aproximadamente 100 personas, en el que está usted incluido, comenzará el experimento a la vez. Cada día, al que nos referiremos como "ronda", recibirá usted 10 puntos. A partir de ahí tendrá que decidir cuántos de esos puntos quiere guardarse para usted y cuántos quiere contribuir a un fondo común. Las contribuciones de todos los participantes al fondo común se sumarán y todos los jugadores, independientemente de lo que hayan contribuido, recibirán un 10% de dicha cantidad total. Sus ganancias al final de cada ronda serán, por tanto, los puntos que usted haya decidido quedarse, más un 10% de la suma de los puntos aportados por todos los jugadores, incluido usted.

Como ejemplo, imagine que la suma de las contribuciones al fondo común ha sido 300 puntos. Cada persona de las 100 que participan recibe un 10% de esta cantidad, es decir, 30 puntos. A ello unirá usted la cantidad que se haya guardado y no haya puesto en el fondo común. Recuerde que al principio de cada ronda recibe usted 10 puntos. Si en este caso en concreto puso usted 4 puntos en el fondo común, sus ganancias totales de la ronda serán 30 (su participación en los beneficios del fondo común) + 6 (la parte que usted no destinó al fondo común), es decir, 36 puntos.

Como ya hemos dicho, tendrá usted que tomar una decisión diaria durante un máximo de 30 días. Las decisiones se pueden tomar en cualquier momento entre las 00:00 h y las 23:59 de cada día. Si usted olvida decidir un día, el software decidirá automáticamente por usted, contribuyendo al fondo común con una cantidad igual a su contribución anterior más o menos dos puntos, decidido al azar. Por favor, no use esta opción, y considérela sólo como una posibilidad de último recurso para una circunstancia excepcional que le impida por cualquier motivo tomar su decisión.

Como estamos interesados en las decisiones que toman las personas, por favor, tome usted sus decisiones diariamente. Si a lo largo del experimento acumula **tres** veces en las que no toma su decisión, nos veremos obligados a excluirle del experimento y no recibirá ningún pago por su participación, ni los puntos acumulados ni el pago por participar.

Para su comodidad, estas instrucciones estarán a su disposición durante todo el experimento.

(d) Preliminar control answer to control question

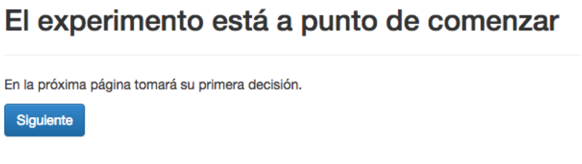

El experimento está a punto de comenzar

En la próxima página tomará su primera decisión.

Siguiente

(e) The experiment is about to start

Supplementary Figure S1: Screens 1 to 5.

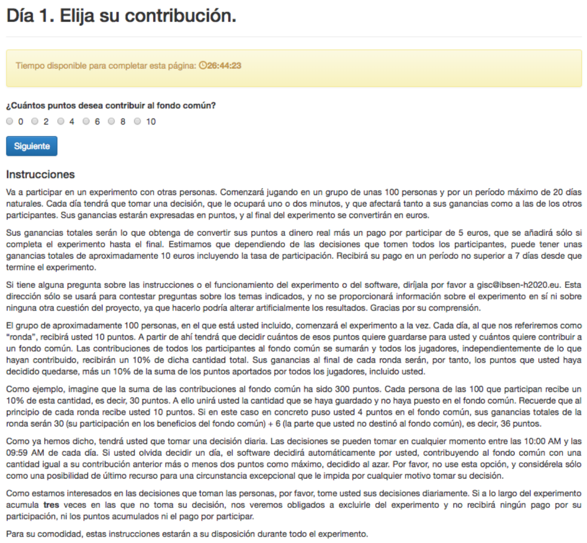

(a) Day 1. Decision screen

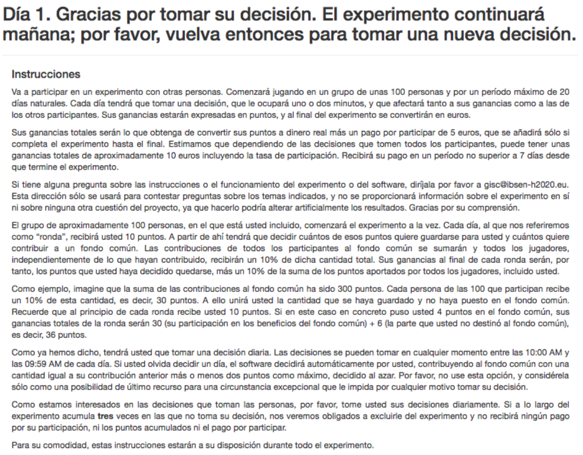

(b) Day 1. After decision screen.

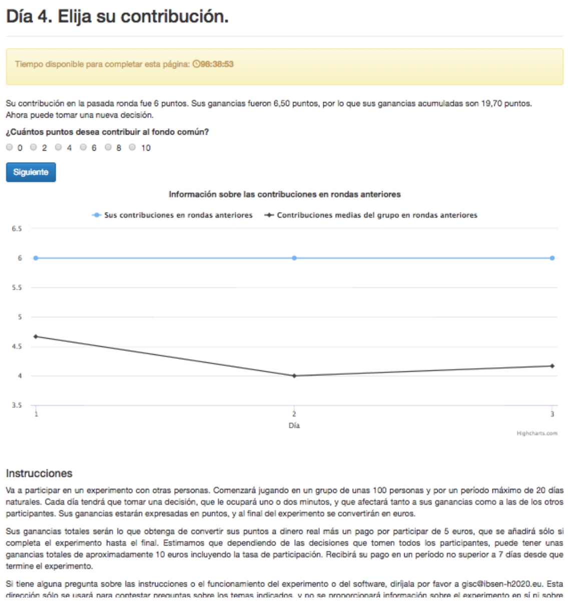

(c) Feedback and decision screen for PGG100 and PGG1000

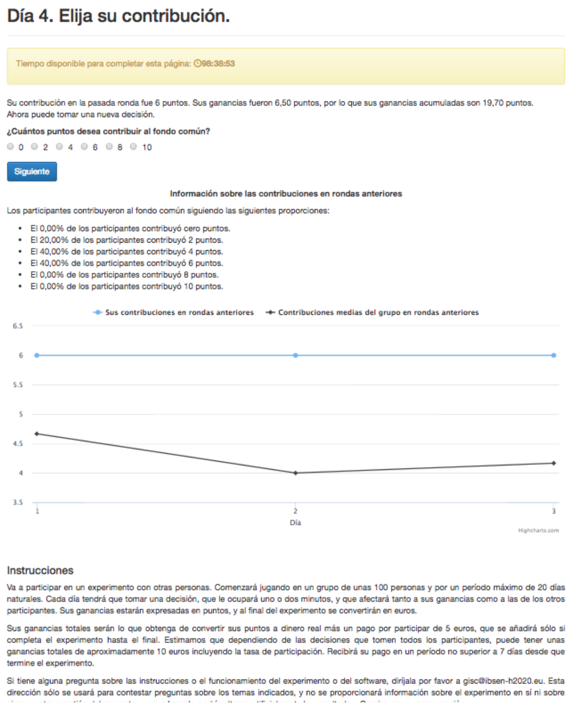

(d) Feedback and decision screen for PGG_HM and PGG_HM2

Supplementary Figure S2: Screens 6 to 9.

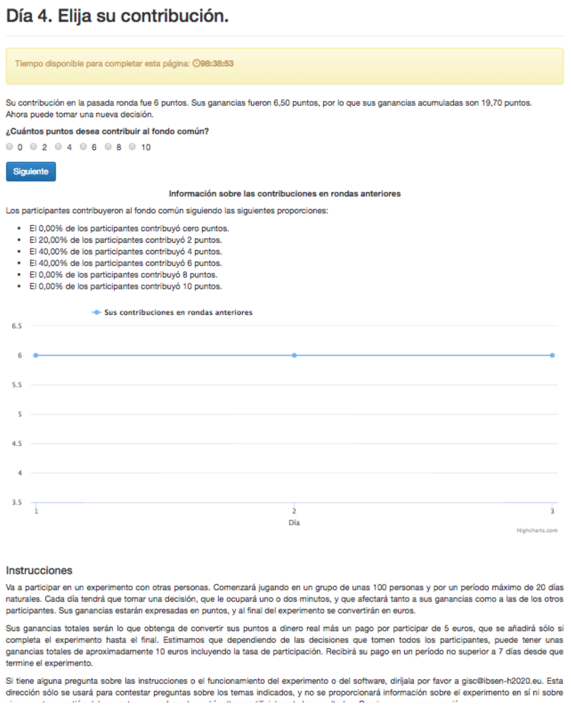

(a) Feedback and decision screen for PGG_H and PGG_H2

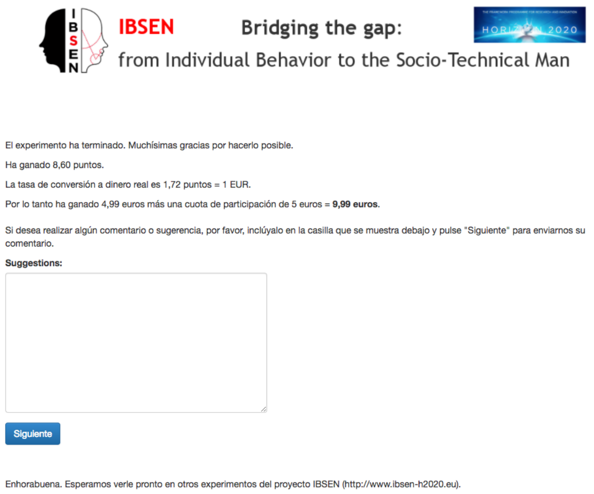

(b) Final results screen

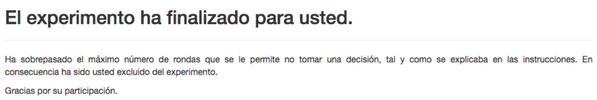

(c) Banned subject screen

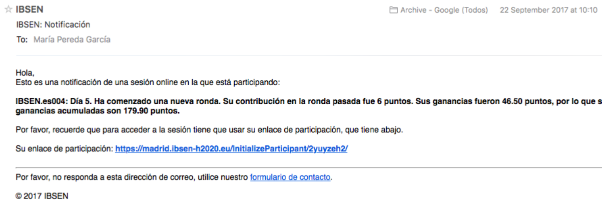

(d) Mail example

Supplementary Figure S3: Screens 10 to 13.

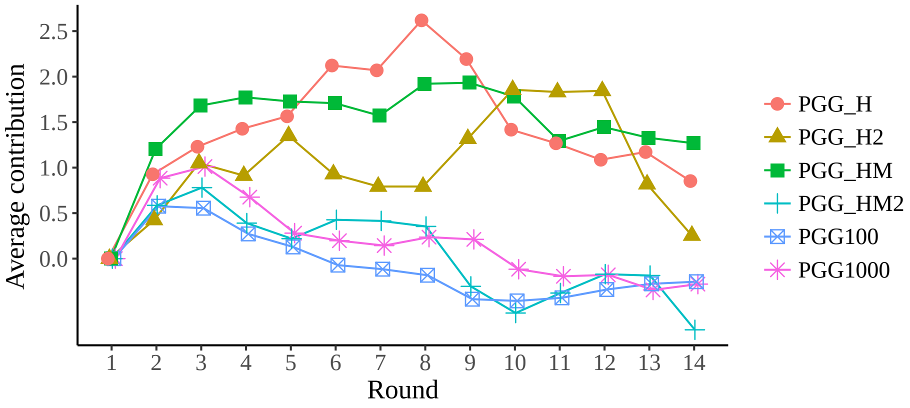

Supplementary Figure S4: Normalised cooperation per treatment. Normalisation is done by subtracting the initial average donation of each treatment from subsequent round values. Error bars are not shown because de distributions of contributions per round are not unimodal; for a representation of dispersion, we point the reader to Fig. 2

2 Supplementary Figures

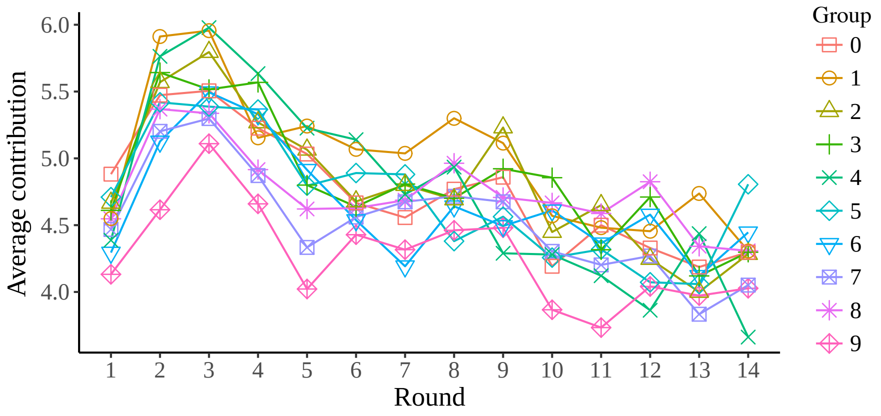

Supplementary Figure S5: Average cooperation values per round and per subsample of 100 subjects randomly chosen among the participants in the PGG1000. Error bars are not shown because de distributions of contributions per round are not unimodal; for a representation of dispersion, we point the reader to Fig. 2

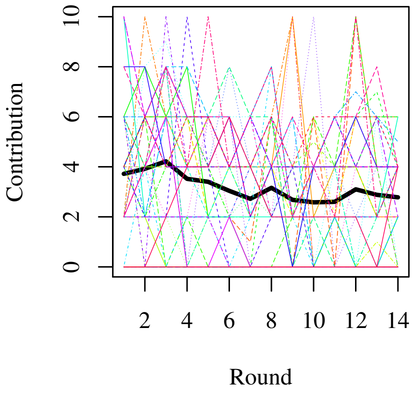

(a) Low contributors: 59%

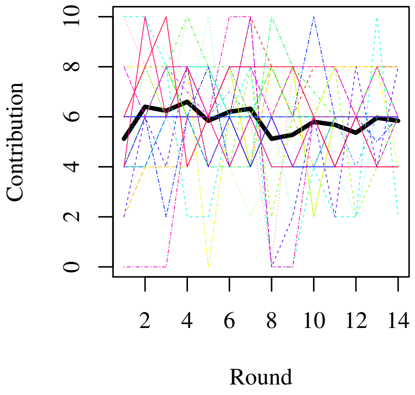

(b) Average contributors: 29%

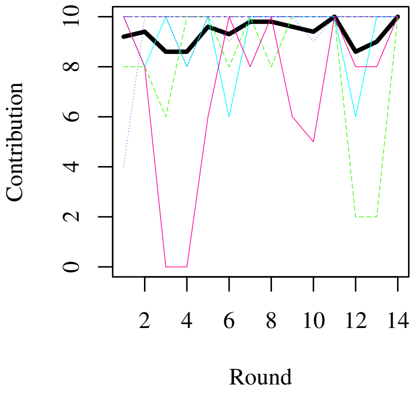

(c) High contributors: 12%

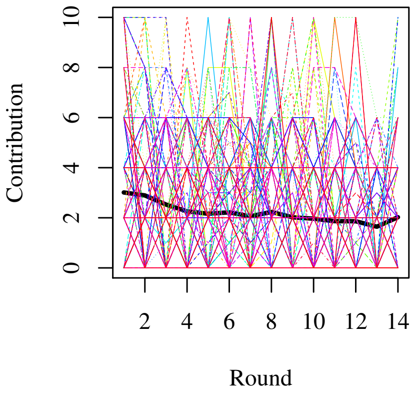

(d) Low contributors: 37%

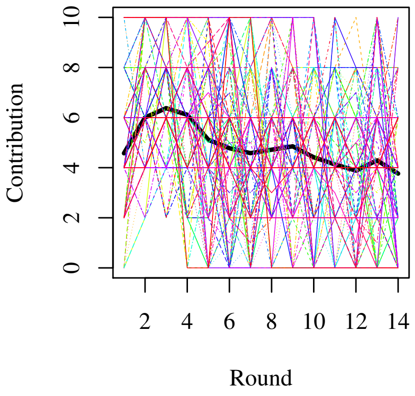

(e) Average contributors: 29%

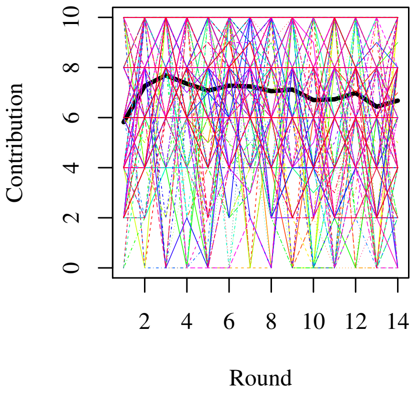

(f) High contributors: 34%

Supplementary Figure S6: PGG100 (top row) and PGG1000 (bottom row) clusters of behaviours. Each coloured line represents the contributions of each participant per round. The black solid line represent the average contribution of the cluster. Each subfigure caption present the percentage of participants for each type of behaviour.

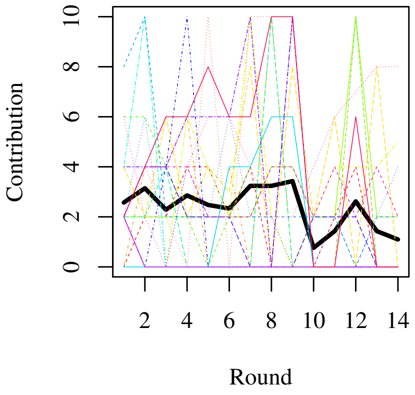

(a) Low contributors: 22%

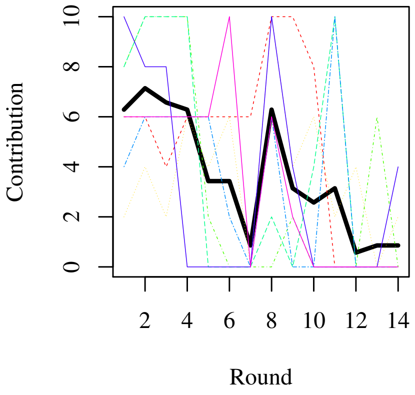

(b) Average contributors: 8%

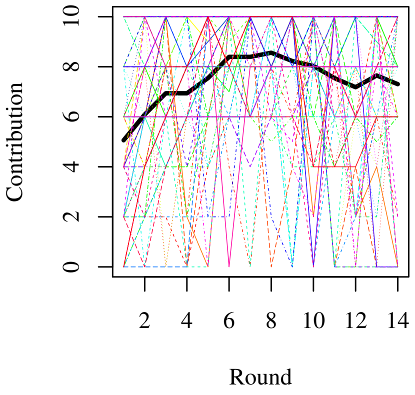

(c) High contributors: 70%

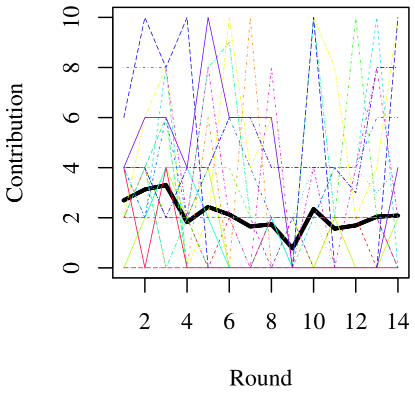

(d) Low contributors: 29%

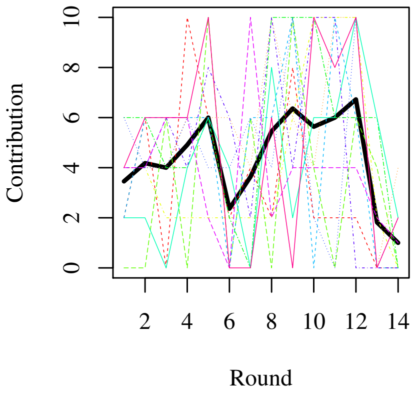

(e) Average contributors: 15%

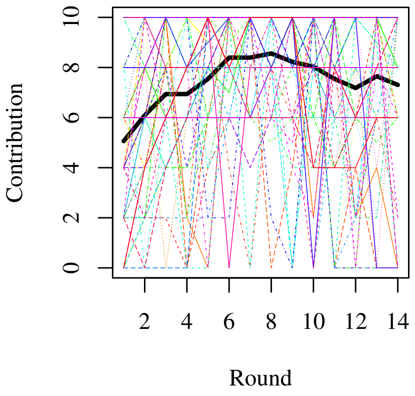

(f) High contributors: 70%

Supplementary Figure S7: PGG_H (top row) and PGG_H2 (bottom row) clusters of behaviours. Each coloured line represents the contributions of each participant per round. The black solid line represent the average contribution of the cluster. Each subfigure caption present the percentage of participants for each type of behaviour.

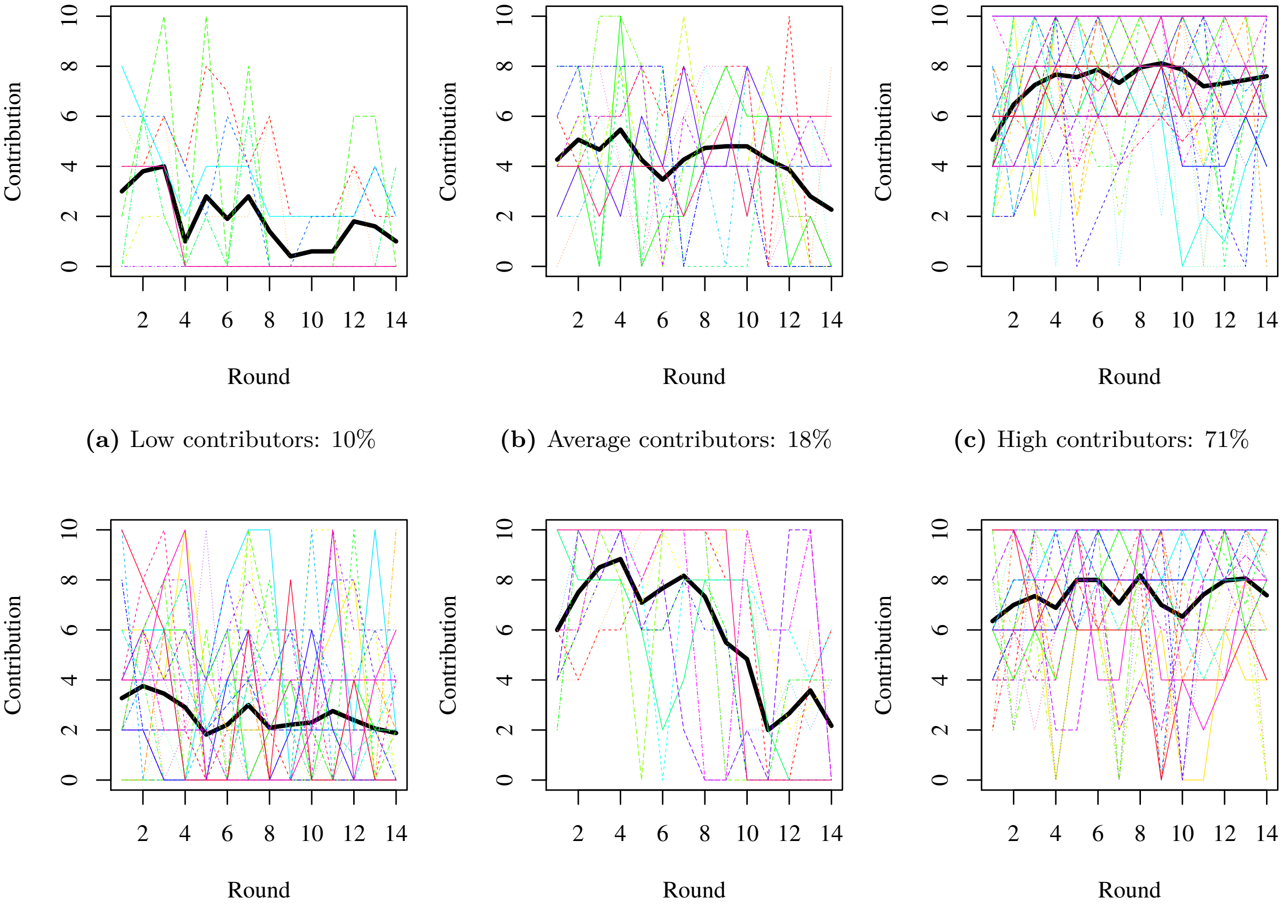

(a) Low contributors: 10%

(b) Average contributors: 18%

(c) High contributors: 71%

(d) Low contributors: 42%

(e) Average contributors: 15%

(f) High contributors: 43%

Supplementary Figure S8: PGG_HM (top row) and PGG_HM2 (bottom row) clusters of behaviours. Each coloured line represents the contributions of each participant per round. The black solid line represent the average contribution of the cluster. Each subfigure caption present the percentage of participants for each type of behaviour.

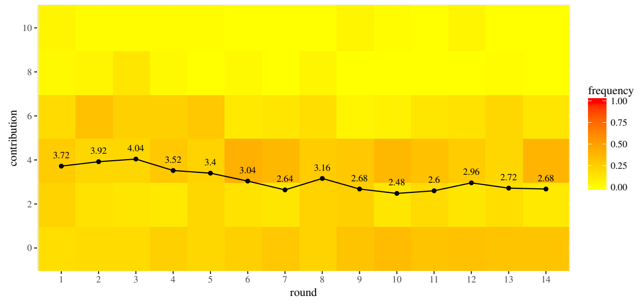

(a) Low contributors: 59%

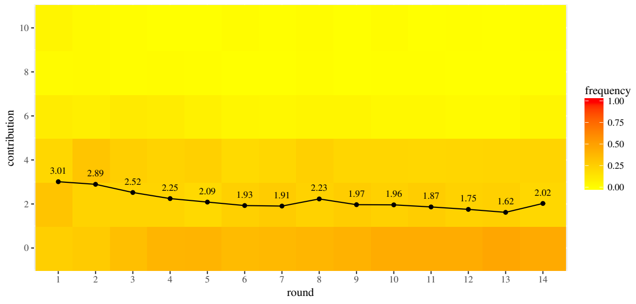

(b) Low contributors: 37%

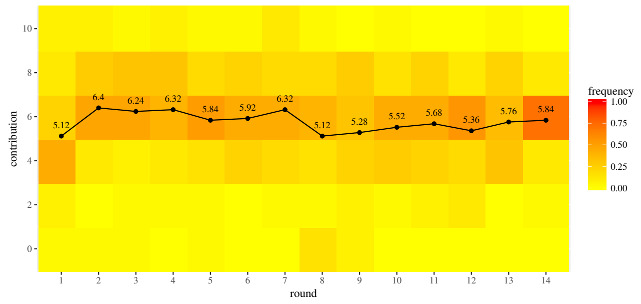

(c) Average contributors: 29%

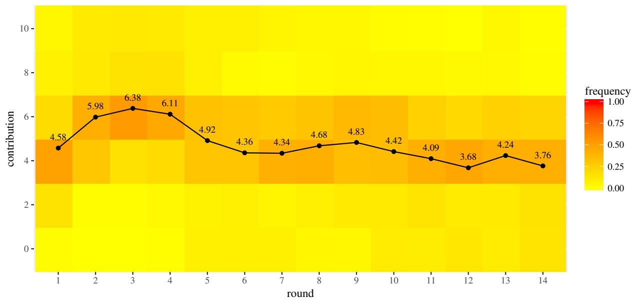

(d) Average contributors: 29%

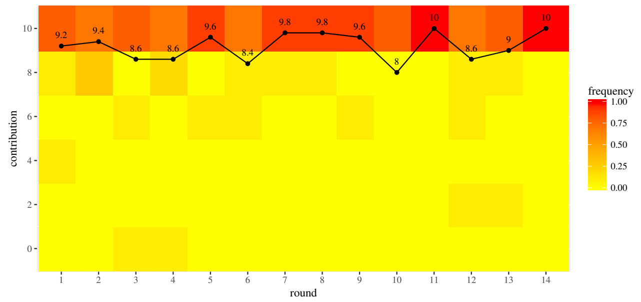

(e) High contributors: 12%

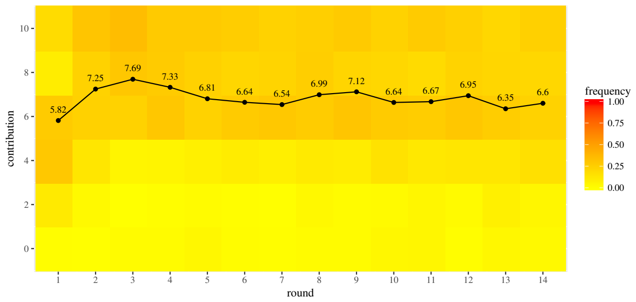

(f) High contributors: 34%

Supplementary Figure S9: Evolution of the frequency of different decisions per round (heatmap) along with the average contribution (black line), for the three types of behaviours in (left) PGG100, and (right) PGG1000. In the heatmap, yellowish (redish) squares correspond to low (high) frequencies (see scale).

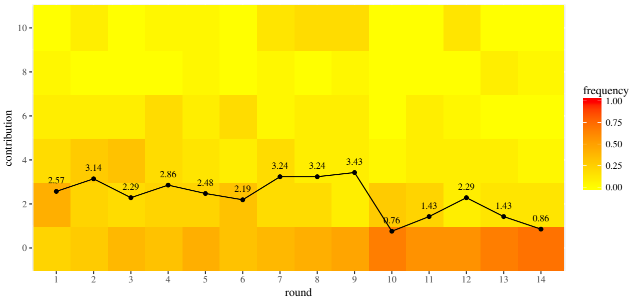

(a) Low contributors: 22%

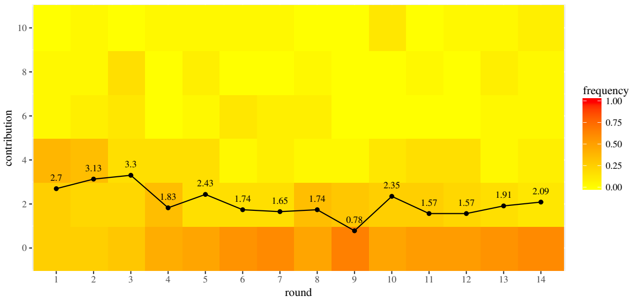

(b) Low contributors: 29%

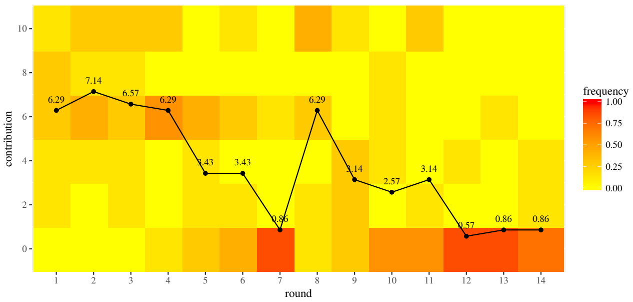

(c) Average contributors: 8%

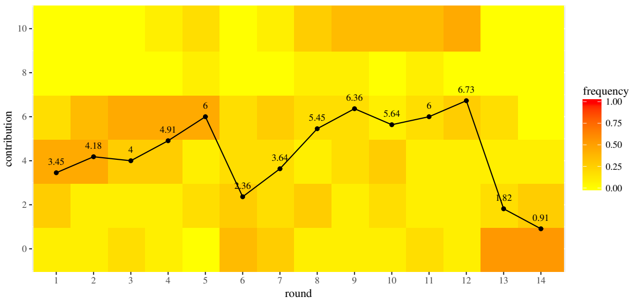

(d) Average contributors: 15%

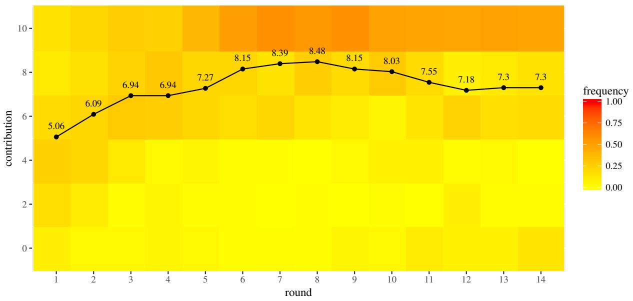

(e) High contributors: 70%

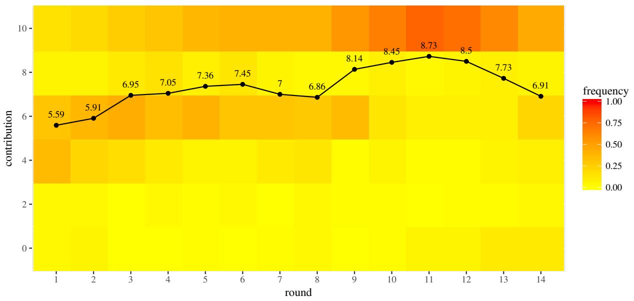

(f) High contributors: 70%

Supplementary Figure S10: Evolution of the frequency of different decisions per round (heatmap) along with the average contribution (black line), for the three types of behaviours in (left) PGG_H, and (right) PGG_H2. In the heatmap, yellowish (redish) squares correspond to low (high) frequencies (see scale).

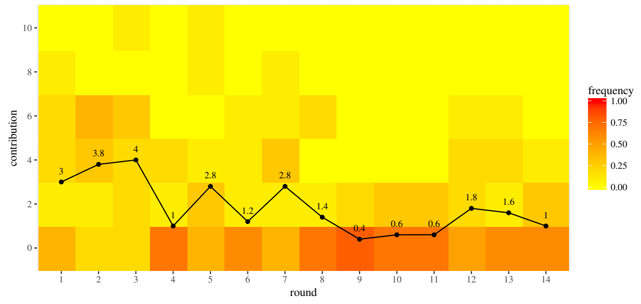

(a) Low contributors: 10%

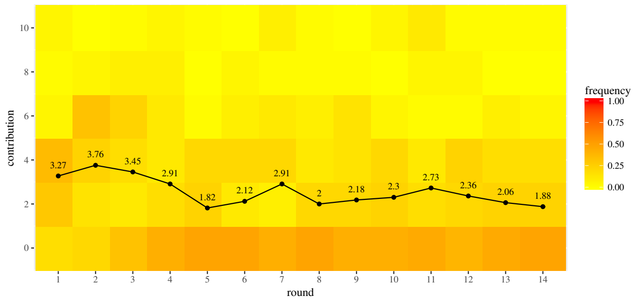

(b) Low contributors: 42%

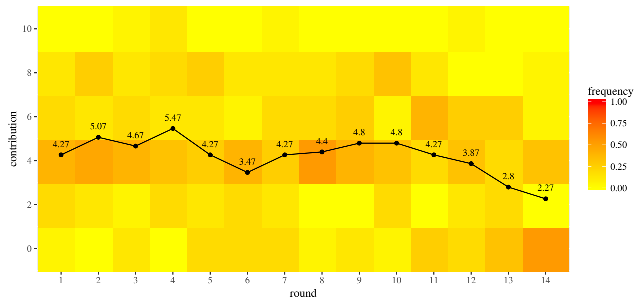

(c) Average contributors: 18%

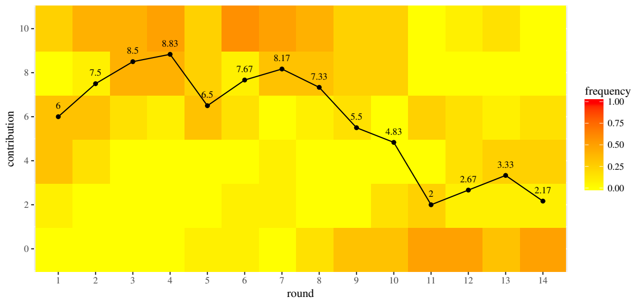

(d) Average contributors: 15%

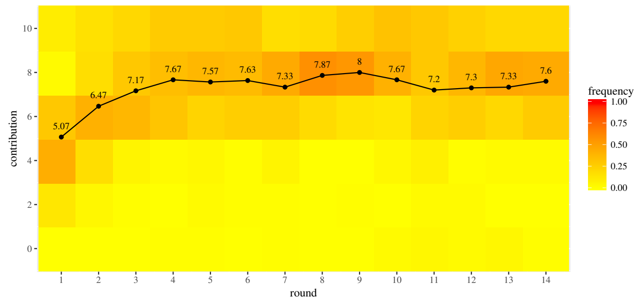

(e) High contributors: 71%

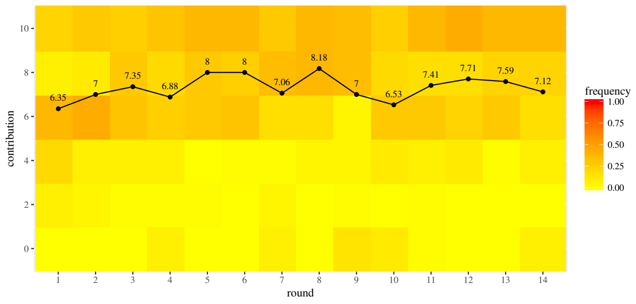

(f) High contributors: 43%

Supplementary Figure S11: Evolution of the frequency of different decisions per round (heatmap) along with the average contribution (black line), for the three types of behaviours in (left) PGG_HM, and (right) PGG_HM2. In the heatmap, yellowish (redish) squares correspond to low (high) frequencies (see scale).

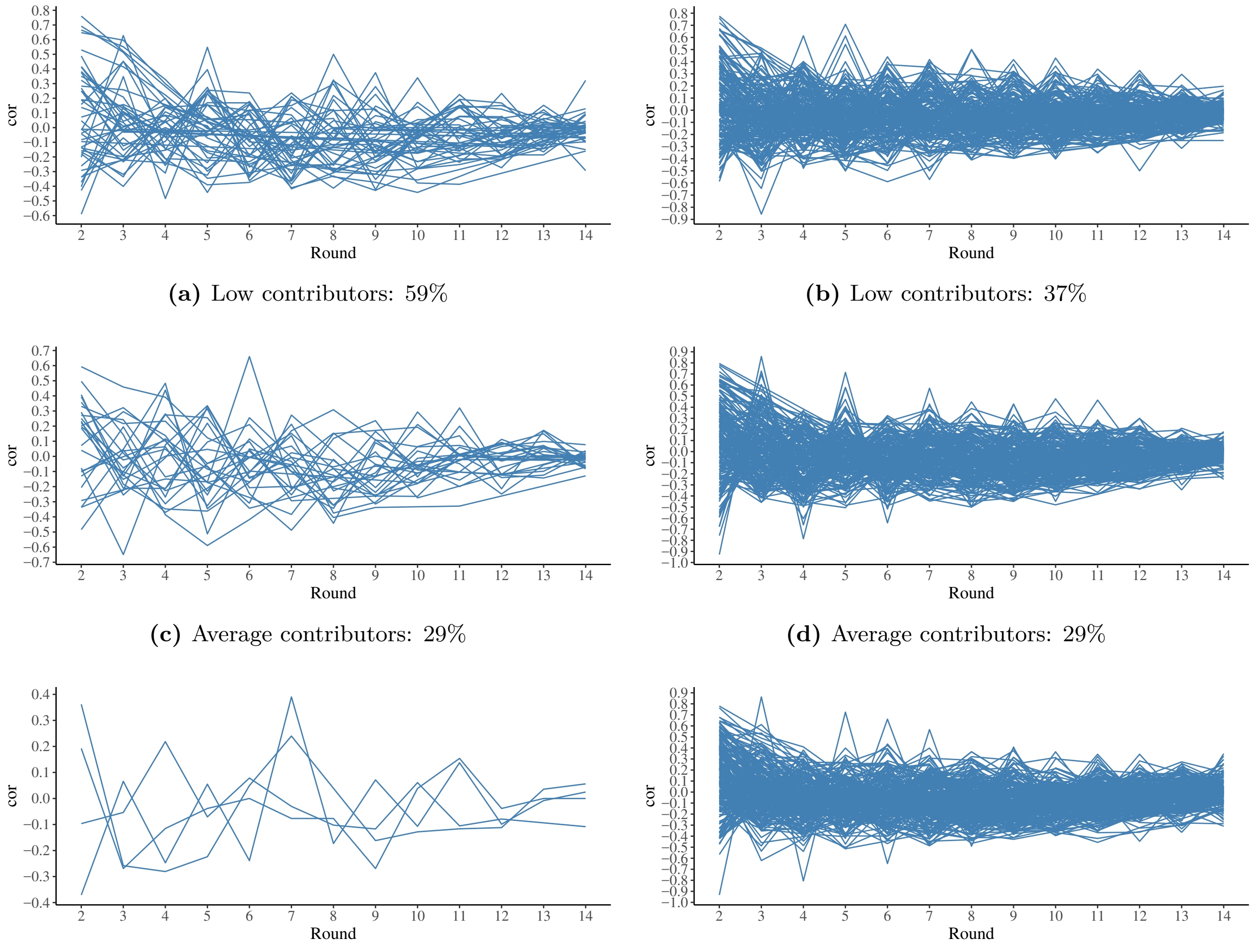

(a) Low contributors: 59%

(b) Low contributors: 37%

(c) Average contributors: 29%

(d) Average contributors: 29%

(e) High contributors: 12%

(f) High contributors: 34%

Supplementary Figure S12: Autocorrelation of individual decisions (round r correlated to round r-1), one line per individual, for the three types of behaviours in (left) PGG100, and (right) PGG1000.

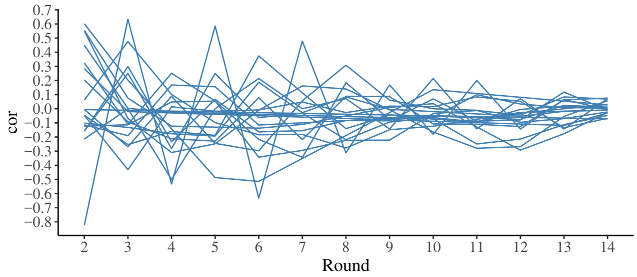

(a) Low contributors: 22%

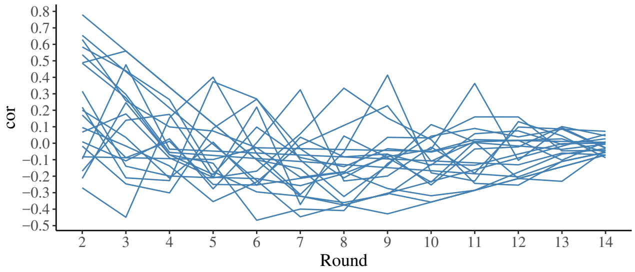

(b) Low contributors: 29%

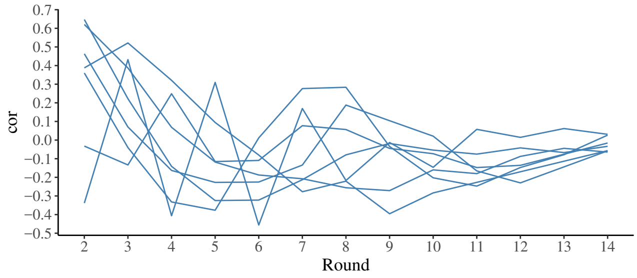

(c) Average contributors: 8%

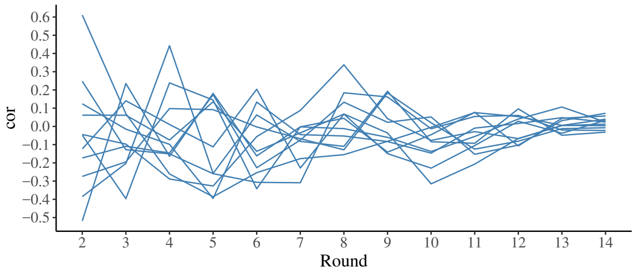

(d) Average contributors: 15%

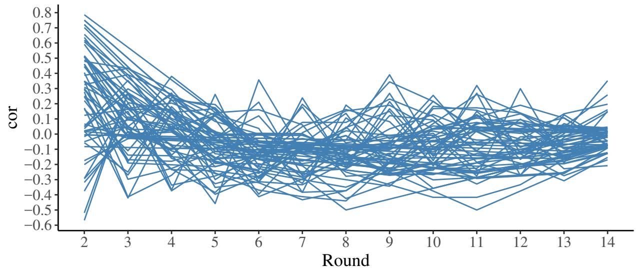

(e) High contributors: 70%

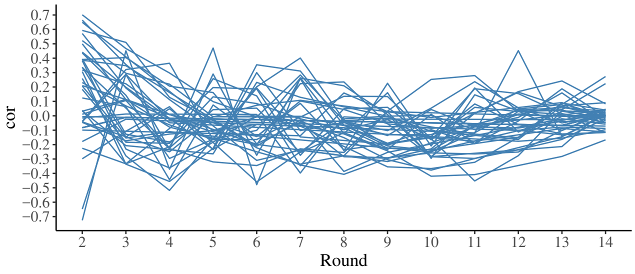

(f) High contributors: 70%

Supplementary Figure S13: Autocorrelation of individual decisions (round r correlated to round r-1), one line per individual, for the three types of behaviours in (left) PGG_H, and (right) PGG_H2.

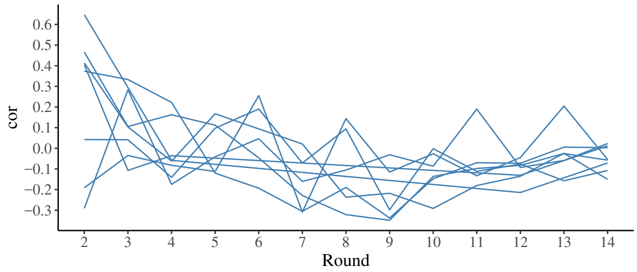

(a) Low contributors: 10%

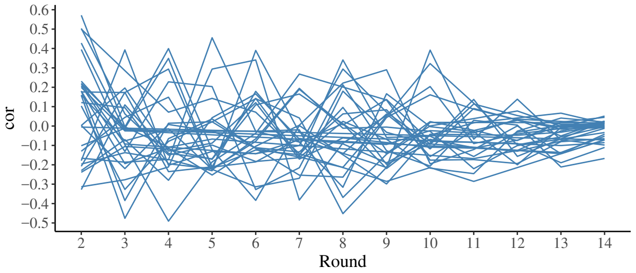

(b) Low contributors: 42%

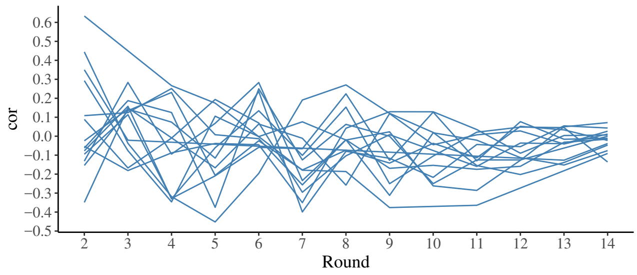

(c) Average contributors: 18%

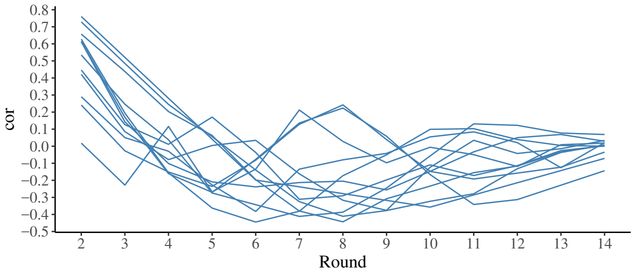

(d) Average contributors: 15%

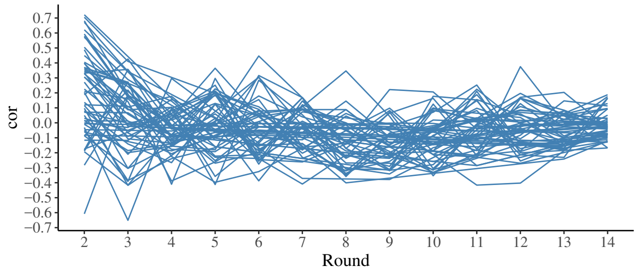

(e) High contributors: 71%

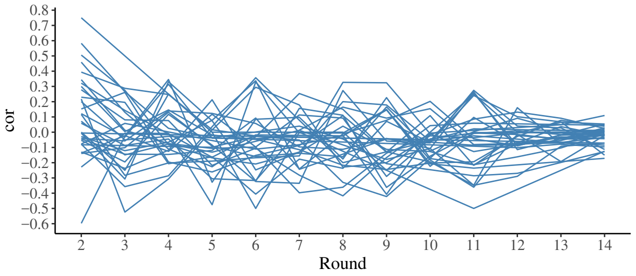

(f) High contributors: 43%

Supplementary Figure S14: Autocorrelation of individual decisions (round r correlated to round r-1), one line per individual, for the three types of behaviours in (left) PGG_HM, and (right) PGG_HM2.

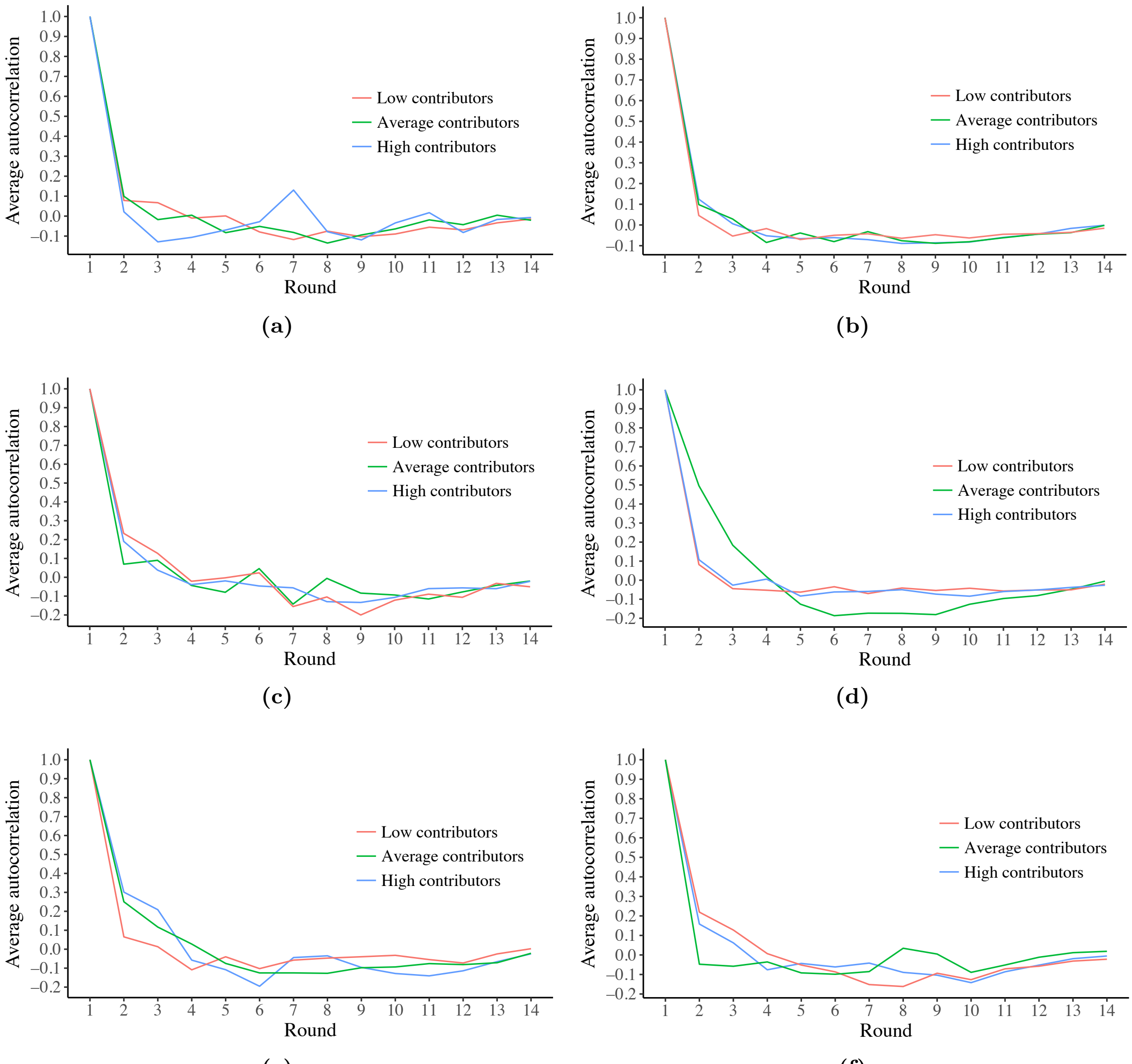

Supplementary Figure S15: Average autocorrelation of individual decisions (round r correlated to round r-1) for the 3 types of behaviours, for (a) PGG100, (b) PGG1000, (c) PGG_HM, (d) PGG_HM2, (e) PGG_H, (f) PGG_H2. I

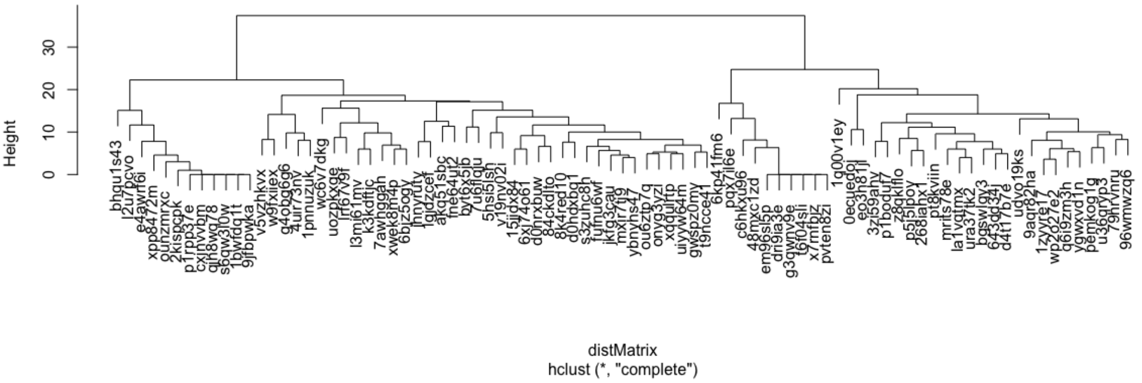

Supplementary Figure S16: Dendrogram of PGG100 hierarchical clustering. Each leaf (bottom) node represents an individual.

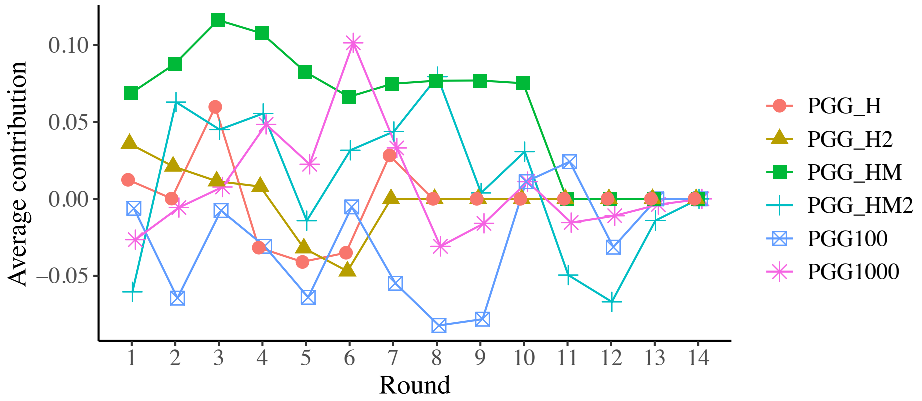

Supplementary Figure S17: Difference between average cooperation values of all the participants (Fig.1) and average cooperation values of those who finished the experiment, per round and per treatment.

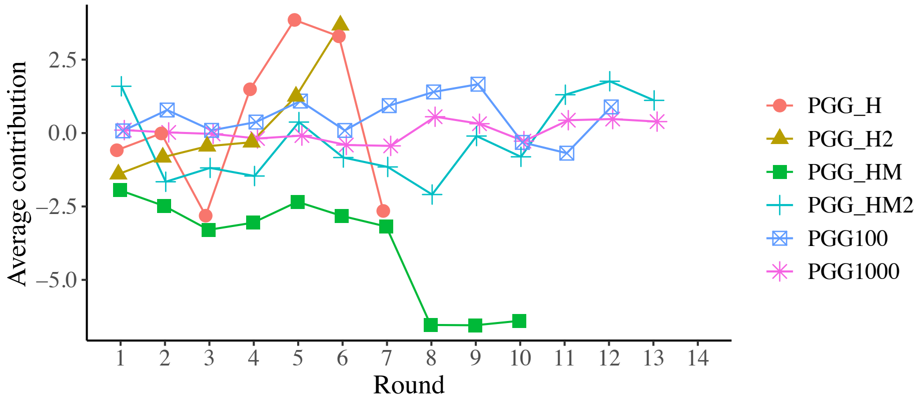

Supplementary Figure S18: Difference between average cooperation values of all the participants (Fig.1) and average cooperation values of those who dropped out, per round and per treatment.

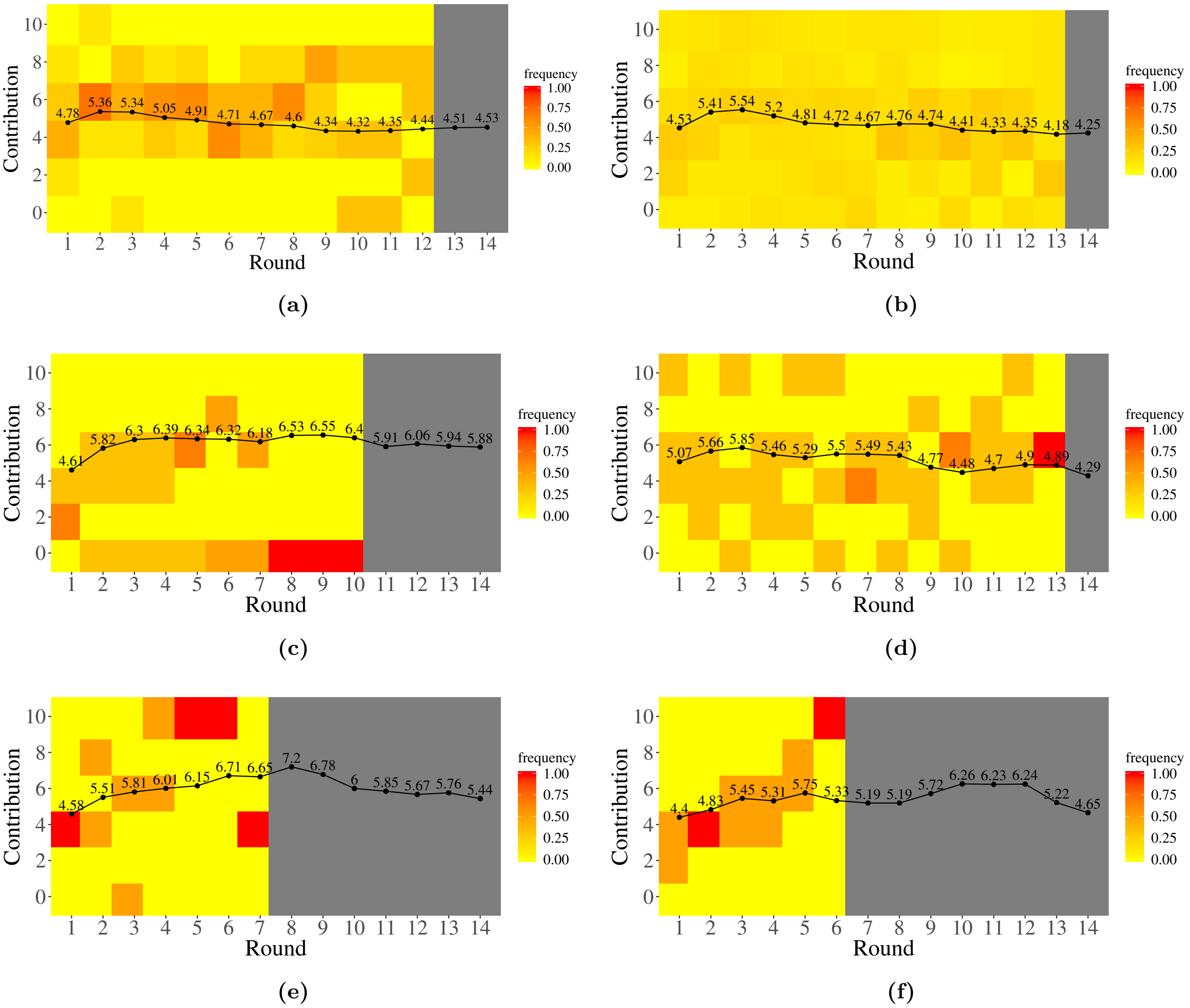

Supplementary Figure S19: Evolution of the frequency of different decisions of people that did not complete the experiment, per round along with the average contribution of the group includong people that complete the experiment (black line), for (a) PGG100, (b) PGG1000,(c) PGG_HM, (d) PGG_HM2, (e) PGG_H, (f) PGG_H2. In the heatmap, yellowish (redish) squares correspond to low (high) frequencies (see scale). In grey, no more people dropped out.
